# Prediction of daily global solar radiation in different climatic conditions using metaheuristic search algorithms: a case study from Türkiye

**DOI:** 10.1007/s11356-024-33785-x

**Published:** 2024-06-19

**Authors:** Hüseyin Bakır

**Affiliations:** Department of Electronics and Automation, Vocational School, Dogus University, Istanbul, 34775 Türkiye

**Keywords:** Daily solar radiation prediction, Climatic and atmospheric measurement parameters, Solar radiation potential of Türkiye, Metaheuristic optimization algorithms

## Abstract

Today’s many giant sectors including energy, industry, tourism, and agriculture should closely track the variation trends of solar radiation to take more benefit from the sun. However, the scarcity of solar radiation measuring stations represents a significant obstacle. This has prompted research into the estimation of global solar radiation (GSR) for various regions using existing climatic and atmospheric parameters. While prediction methods cannot supplant the precision of direct measurements, they are invaluable for studying and utilizing solar energy on a global scale. From this point of view, this paper has focused on predicting daily GSR data in three provinces (Afyonkarahisar, Rize, and Ağrı) which exhibit disparate solar radiation distributions in Türkiye. In this context, Gradient-Based Optimizer (GBO), Harris Hawks Optimization (HHO), Barnacles Mating Optimizer (BMO), Sine Cosine Algorithm (SCA), and Henry Gas Solubility Optimization (HGSO) have been employed to model the daily GSR data. The algorithms were calibrated with daily historical data of five input variables including sunshine duration, actual pressure, moisture, wind speed, and ambient temperature between 2010 and 2017 years. Then, they were tested with daily data for the 2018 year. In the study, a series of statistical metrics (*R*^2^, MABE, RMSE, and MBE) were employed to elucidate the algorithm that predicts solar radiation data with higher accuracy. The prediction results demonstrated that all algorithms achieved the highest *R*^2^ value in Rize province. It has been found that SCA (MABE of 0.7023 MJ/m^2^, RMSE of 0.9121 MJ/m^2^, and MBE of 0.2430 MJ/m^2^) for Afyonkarahisar province and GBO (RMSE of 0.8432 MJ/m^2^, MABE of 0.6703 MJ/m^2^, and R^2^ of 0.8810) for Ağrı province are the most effective algorithms for estimating GSR data. The findings indicate that each of the metaheuristic algorithms tested in this paper has the potential to predict daily GSR data within a satisfactory error range. However, the GBO and SCA algorithms provided the most accurate predictions of daily GSR data.

## Introduction

The Russia-Ukraine war has clearly shown how important a threat energy security is to a country’s economy (Guchua and Jomidava 2023; Liu et al. 2023). Countries with high energy dependence are susceptible to the effects of such crises, even if they are not directly involved in the war. All these are not historical events but are occurring in today’s world. The war has highlighted the importance of energy diversity for decision-makers, prompting a re-evaluation of energy policies and the recognition of the vital role of diversity in energy production (Xin and Zhang 2023). On the other hand, energy dependency is undoubtedly inevitable for countries that do not have their own fossil resource reserves. However, governments have once again acknowledged that renewable energy represents the most secure option, offering numerous advantages, including the fact that it is free, clean, abundant, reliable, sustainable, and most importantly, unaffected by any crisis, with endless resources (Krane and Idel 2021; Colgan et al. 2023; Kouyakhi 2023). The transition to renewables reduces reliance on imported fossil fuels, improves energy security, and result in lower costs over time. It also contributes to the achievement of the Sustainable Development Goals (SDGs) (Nguyen et al. 2021; Marco-Lajara et al. 2023). The growing interest of countries in achieving net-zero emissions by 2050 further strengthens the case for renewable energy. The orientation of countries to suitable renewable energy sources according to their geographical locations can pull back energy dependency to minimum levels (Ağbulut et al. 2023). The most significant factor influencing a country’s transition to a renewable energy source is the extent of potential that the relevant country has for that renewable energy source and the extent to which it can benefit from it. This is undoubtedly dependent on the geographical location of the country and the technology available therein. Policymakers, researchers, and consumers are all aware of the potential of renewable energy sources and are focusing their research on the feasibility of utilizing them (Said et al. 2022; Marco-Lajara et al. 2023).

Considering all renewable energy sources, solar energy emerges as a particularly noteworthy candidate (Nguyen et al. 2024). Its salient attributes include the generation of clean, plentiful, and easy electricity (Said et al. 2022). Solar thermal (Pang et al. 2020) and solar photovoltaic (PV) (Sharma et al. 2022) represent the two principal avenues for harnessing the sun’s energy for our needs. Solar photovoltaics has recently become a major driver of renewable energy growth. The most recent report published by the International Energy Agency (IEA) indicates that three-quarters of all new renewable energy capacity additions globally were attributable to solar PV in 2023 (IEA 2023). In comparison to other renewables such as hydroelectric or geothermal, solar panels have a relatively simple technological design. Consequently, their installation and maintenance are relatively straightforward (Awasthi et al. 2020). Solar energy systems can be scaled to fit various needs (Hoang and Nguyen 2021). For instance, solar farms can produce electricity for entire communities, while small-scale rooftop panels can power a home. Most importantly, solar energy is the source of many other energy forms on Earth (Guermoui et al. 2020). It is also considered one of the most promising renewable energy resources for meeting the world’s energy demand at a considerable rate (Belmahdi and Bouardi 2024).

Global solar radiation (GSR) plays a pivotal role in numerous facets of human existence. Many significant sectors including electricity production, agriculture, and tourism are directly dependent on the amount of GSR (Zhou et al. 2021; Soomar et al. 2022). Accordingly, the investors have continuously tracked and revised their plans, and future investments in these sectors to take more benefits from solar radiation. However, solar radiation varies considerably due to its dependence on synoptic and local weather patterns, which presents a challenge in accurately estimating solar radiation data (Rodríguez-Benítez et al. 2020). The unpredictability of solar radiation and the variable output of solar PV systems can negatively affect the supply–demand balance (Gianfreda et al. 2016). This case can also led to less or more electricity production than the amount to be produced especially in the intra-day electricity market from solar energy systems, thus significantly reducing the profit margin (Ağbulut 2022). Therefore, it is of the utmost significance to accurately predict the solar radiation data for the successful operation and management of solar power plants and to take more benefit from the sun. Accurate solar radiation prediction represents a significant breakthrough in the energy sector, leading to more efficient power generation, grid management, and energy trading (Shah et al. 2021; Jumin et al. 2021).

Even though solar energy is available in abundance, its quantity and concentration vary considerably from one region to another. A continuous, robust, and reliable measurement of solar radiation data is not available for all regions, even in the most developed countries of the world. The primary reason for this is that the cost of solar radiation measurement devices is exceedingly high, and their maintenance is challenging. For example, approximately 10% of weather stations in China are equipped to record solar radiation data (Fan et al. 2018b), while 7% of those in Türkiye can do so (Ağbulut et al. 2021). These figures demonstrate the challenge of accessing solar radiation data, which is crucial for the sustainable production of major sectors.

It is possible to both set a relationship and predict the amount of renewable energy sources in a given region since there are strong interactions between renewable energy sources and climatic parameters. This is a highly beneficial tool for researchers engaged in the study of renewable energy, as it enables them to observe the renewable energy output of a given region by utilizing the climatic parameters specific to that region. This case has prompted researchers to investigate methods for obtaining solar radiation data in a more accessible, precise, continuous, and robust manner. In recent years, researchers have focused on forecasting GSR data by using machine learning approaches (Jathar et al. 2024), empirical models (Gürel et al. 2020), deep learning algorithms (Bamisile et al. 2022), time series (Peñalva et al. 2022), support vector machine (Ghimire et al. 2022), artificial neural network algorithms (Geetha et al. 2022), fuzzy logic (Patel et al. 2022), k-nearest neighbour (Duarte et al. 2022), random forest (Srivastava et al. 2019), ensemble learning (Cannizzaro et al. 2021), intelligence models (Tao et al. 2021), etc. The majority of researchers have sought to accurately predict GSR data in conjunction with readily recordable climatic and atmospheric data for a specific region or country. Table 1 provides an overview of the literature on the prediction of GSR data.

**Table 1:** A brief of literature works on the estimation of solar radiation data

References, year	Method	Location	Input variables	Forecast variable	Forecast horizon	Data scale	Evaluation metrics	Best performing model (s)
Mousavi et al. (2017)	ANN/SA, ANN, and SVM	Iran (Mashhad province)	Wind speed, atmospheric pressure, minimum and maximum temperatures, earth skin temperature, average air temperature, and relative humidity	GSR	Daily	From 1995 to 2014	RMSE, MAE, and *R*^2^	ANN/SA
Khosravi et al. (2018)	SVR, ANFIS, FIS, MLFFNN, and RBFNN	Abu Musa Island, Iran	Local time (hour), humidity, pressure, wind speed, temperature	GSR	Hourly	Past few months or years	*R*, RMSE	SVR and MLFFNN
Zhang et al. (2019)	PSO-BP, BP, and Six statistical models	Northwest China	Sunshine duration, extraterrestrial radiation, relative humidity, minimum and maximum temperatures	GSR	Daily	From 1997 to 2016	*R*^2^, RMSE, and MAE	PSO-BP
Jiang et al. (2019)	ResnetTL, ResnetO, and ANN_P	China	Measured solar radiation values from 90 Chinese radiation stations	GSR	Hourly	January 01, 2007, to December 31, 2008	RMSE, MBE, and *R*^2^	ResnetTL
Fan et al. (2019)	72 empirical models	China	Meteorological data	DSR	Daily Monthly	From 1966 to 2015	MABE, RMSE, *R*^2^, GPI, NRMSE, U_95_, and *t*-statistics	Daily: VIII-3, IV-3, IV-3, IV-3, and III-2 Monthly: IV-3, VIII-3, VII-7, VIII-5, and IV-5
Feng et al. (2020)	PSO-ELM, M5T, GRNN, ELM, AE, and SVM	Loess Plateau, China	Five daily meteorological variables	GSR	Daily	1961–2016	rRMSE, NS, MAE	PSO-ELM
Fan et al. (2020)	SVM, SVM-BAT, SVM-WOA, SVM-PSO, MARS and XGBoost	China	Meteorological data	DSR	Daily	January 2014 to March 2017	*R*^2^, MAE, RMSD, and SI	SVM-BAT
Ağbulut et al. (2021)	k-NN, ANN, SVM, and DL	Provinces of Türkiye **(**Kırklareli, Tokat, Nevşehir and Karaman)	Extraterrestrial solar radiation, day length, cloud cover, minimum and maximum temperatures	GSR	Daily	January 01, 2018, to December 31, 2019	MABE, MBE, MAPE, RMSE, *R*^2^, rRMSE, and t-stat	ANN
Alrashidi et al. (2021)	KNN, DT, ANN, RF, SVR, SVR-GOA-BA_K_	Provinces of Saudi Arabia (Dhahran, Riyadh, and Jeddah)	Time-related variables, climate-related variables, and one-hour lag observations of DHI, GHI, and DNI	GSR	Hourly	June 1, 2013, to May 31, 2017	nRMSE, nMAE, MAPE, MAE, RMSE, and *R*^2^	SVR-GOA-BA_K_
Bounoua et al. (2021)	ANN, tree-based ensemble methods, and 22 empirical models	Five regions in Moroccan (Erfoud, Tan-Tan, Missouri, Oujda, and Zagora)	Air vapour pressure deficit, wind speed, daily temperature gradient, mean air temperature, mean relative humidity, daily horizontal top of atmosphere solar irradiance	GSR	Daily	August 18, 2011, to September 30, 2015	nRMSE, nMAE, and *R*	Random Forest
Jumin et al. (2021)	BDTR, LR, NNBN, and NNGN	Province of Malaysia (Kuala Terengganu)	Historical solar radiation data	GSR	Hourly	2008 (March and April), 2009 (January, February, March, and April), and 2010 (April)	*R*, *R*^2^, RAE, RMSE, and RSE	BDTR
Tao et al. (2021)	ANFIS, ANFIS-SSA, ANFIS-muSG, ANFIS-GWO, ANFIS-DA, ANFIS-PSO, ANFIS-GOA, and ANFIS-GA	North Dakota, USA	Air temperature data	GSR	Daily	From 2010 to 2018	MARE, MAE RMSRE, AAPRE, RMSE, and *R*^2^	ANFIS-muSG
Huang et al. (2021)	12 machine learning models	Ganzhou City, China	Air pressure, sunshine duration, precipitation, visibility, humidity, wind speed, and temperature	GSR	Daily Monthly	1980–2016	*R*^2^, MAE, RMSE	XGBoost
Bamisile et al. (2022)	ANN, CNN, RNN, SVR, RF, and PR	Four locations in Nigeria (Borno, Kano, Yobe, and Zamfara)	Sun height, ambient temperature, wind speed, year, month, day, hour	GSR and DSR	Hourly	Hourly data between 2005 and 2016	*r*, RMSE, NMBE, and MAE	RNN
Qiu et al. (2022)	74 existing and 4 new empirical models	China	Temperature data	GSR	Daily	From 1967 to 2016	d_IA_, MBE, *R*^2^, MAE, RMSE, RRMSE	N1-4 model
Jamei et al. (2022)	LSSVM-ISA, MARS, GRNN, MLRI	Iran (Khuzestan province)	Sunshine hours, relative humidity, average daily temperature, average wind velocity, day of the year	GSR	Daily	July 01, 2009, to July 01, 2019	RMSE, MAPE, *R*, and NS	LSSVM-ISA
Nematchoua et al. (2022)	Six machine learning algorithms (LM, DT, SVM, DL, RF and GBT)	Twenty-seven European countries	Relative humidity, global solar radiation, wind speed, and daily data of air temperature	GSR	Daily	Two periods (1960–1990) and (2000–2019)	RMSE, ARE, AAE, and R^2^	SVM
Woldegiyorgis et al. (2022)	ANN, Three sunshine empirical models (AP, LO, and GM)	Ethiopia	Wind speed, relative humidity, pressure, minimum, and maximum temperatures	GSR	Daily	January 01, 2014, to December 31, 2016	MBE, RMSE, and *R*^2^	ANN
Patchali et al. (2022)	20 simple empirical models	Togo	Relative humidity, sunshine duration, and air temperature	GSR	Daily	1990–1992	PME, ME, RMSE, NSE, and *r*	M13 and M18
Demir and Citakoglu (2023)	ELM, LSTM, GPR, KNN, and SVMR	Türkiye	Latitude, wind speed, month, year, elevation, longitude, relative humidity, temperature	GSR	Monthly	1967–2020	*R*^2^, NSE, RMSE, MAE, MARE,	LSTM
Belmahdi and Bouardi (2024)	MLP, FFBP, ARIMA, LR, RBFNN, RF, and GPR	Tetuan and Tangier locations in Morocco	Geographical, astronomical, computational, and meteorological data	GSR	Hourly	2012–2015	MAPE, *R*^2^, MAE, MSE	RF
**The present study**	**GBO, HHO, BMO, SCA, and HGSO**	**Provinces of Türkiye (Afyonkarahisar, Rize and Ağrı)**	**Sunshine duration, actual pressure, moisture, wind speed, and ambient temperature**	**GSR**	**Daily**	**January 01, 2010, to December 31, 2018**	***R***^**2**^**, MBE, MABE, and RMSE**	**GBO and SCA**

As can be seen from the previous works presented in Table 1, there is no single algorithm that can predict with high accuracy the GSR data for any region. In other words, one algorithm may present the optimal GSR prediction result for a given region, yet simultaneously yield the poor results for another region. From this perspective, testing novel algorithms in solar radiation forecasting would be beneficial. To the best of the authors’ knowledge, there is no published paper regarding the forecasting of daily global solar radiation data of Türkiye using the Gradient-Based Optimizer (GBO), Harris Hawks Optimization (HHO), Barnacles Mating Optimizer (BMO), Sine Cosine Algorithm (SCA), and Henry Gas Solubility Optimization (HGSO), heretofore. To address this gap and observe the performance of these algorithms in solar radiation prediction, three provinces (Afyonkarahisar, Rize, and Ağrı) in Türkiye with varying climatic conditions were selected for analysis. In the study, algorithms are calibrated with sunshine duration, actual pressure, moisture, wind speed, and ambient temperature. The main criteria in the selection of these input variables are their easy-recordable, easy-accessible, and low-cost characteristics in addition to their high correlation with GSR. Subsequently, a series of statistical metrics (*R*^2^, MABE, RMSE, and MBE) are employed to elucidate the algorithm that predicts solar radiation data with greater accuracy.

The remaining sections of the paper are organized as follows. The “Methodology” section gives geographic details of the study sites, a description of the dataset, the basics of metaheuristic algorithms, performance evaluation metrics, and the mathematical model of the daily GSR prediction problem. The “Results and discussion” section elaborates on the forecasting results. Finally, the findings of the study are summarized in the “Conclusions” section.

## Methodology

### Study sites

Türkiye has largely met its energy needs by burning fuel in thermal power plants. However, due to its geographical location, the country has great potential for renewable energy sources. According to Türkiye’s Solar Energy Potential Atlas, published by the Ministry of Energy and Natural Resources (MENR), the average annual total hours of sunshine is 2741 h, and the average annual GSR is 1527.46 kWh/m^2^ (MENR 2022a). Admittedly, the reported data shows why Türkiye is an attractive country for solar energy investment.

**Fig. 1:**
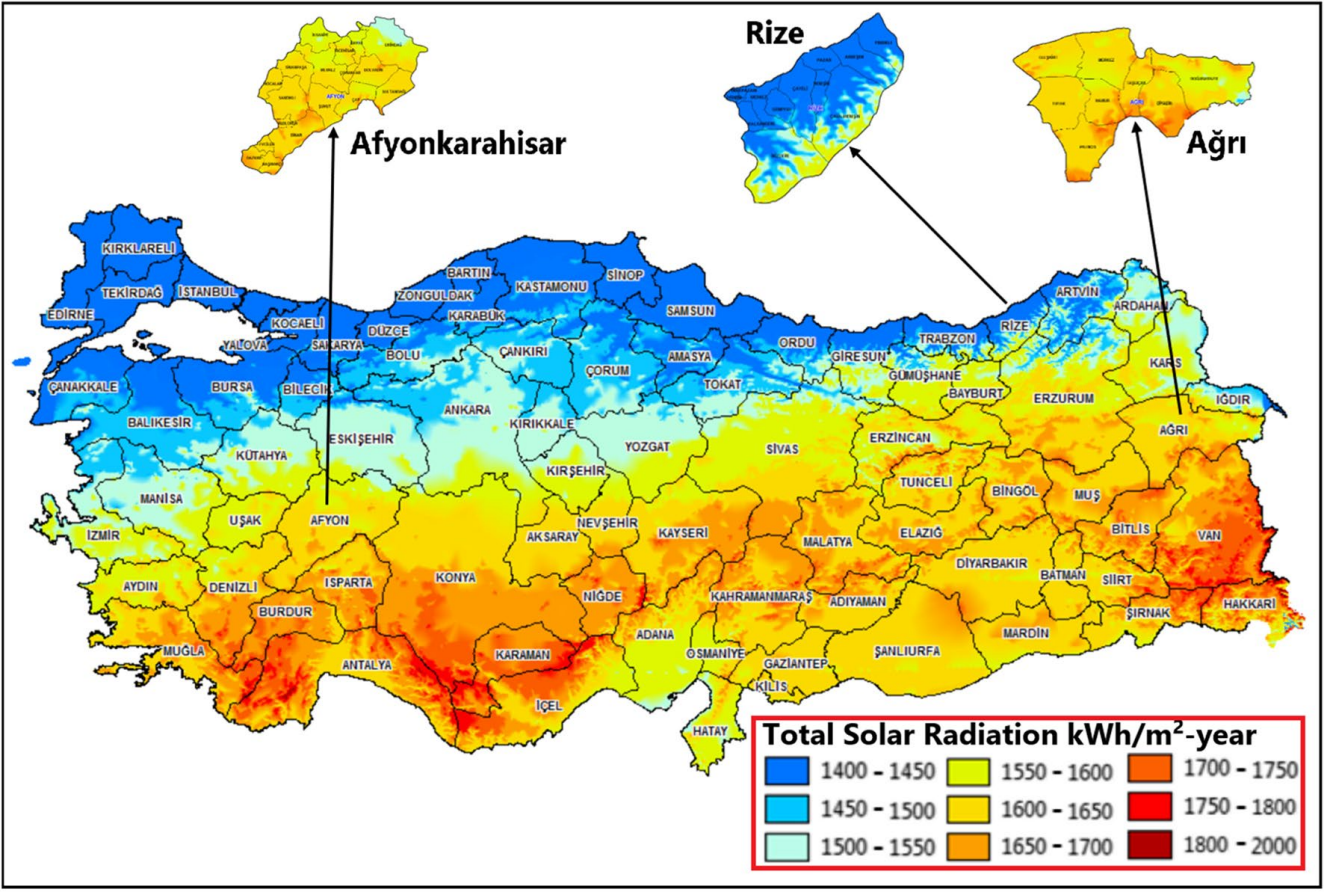
Annual global solar radiation of the Türkiye provinces (MENR 2022b)

Proper site selection for solar energy projects is critical to maximizing energy production and ensuring the economic viability of these projects. Solar radiation, the amount of solar energy received by a particular site, plays a key role in determining the suitability of a site for solar energy development. In the present study, three provinces (Afyonkarahisar, Rize, and Ağrı) in Türkiye are selected for the prediction of daily GSR data. The location of these sites on the map of Türkiye and the annual GSR scale is shown in Fig. 1 (MENR 2022b). The study considers provinces with different scales of solar radiation. From this perspective, the sites selected in this study represent the total solar radiation potential of Türkiye.

### Description of dataset

This research focuses on the forecasting of daily GSR data using different metaheuristic algorithms for three provinces (Afyonkarahisar, Rize, and Ağrı) in Türkiye. In this direction, historical data covering the period from 01 January 2010 to 31 December 2018 are used. The dataset is taken from the Turkish State Meteorological Service (TSMS) (TSMS 2022). Historical data between 2010 and 2017 years are used to train the optimization algorithms. The remaining data (year 2018) is used to test the performance of the algorithms. The algorithms are trained with five input parameters: sunshine duration (hours), actual pressure (hPa), moisture (%), wind speed (m/s), and ambient temperature (°C).

### Optimization framework of metaheuristic algorithms

The optimization procedure of the GBO, HHO, BMO, SCA, and HGSO algorithms is introduced in the following subsections. Similar notations are used for basic parameters (number of design variables, population size, etc.) in the definition of the algorithms to facilitate the reader’s understanding.

#### Gradient-based optimizer (GBO)

GBO is a powerful artificial intelligence algorithm developed by combining the gradient concept and the swarm-based strategy (Ahmadianfar et al. 2020). The algorithm uses two search operators to produce feasible solutions. These are the gradient search rule (GSR) and the local escape operator (LEO). The first of the operators contributes to the exploration ability of the algorithm, while the other provides escaping of the local solution trap (Hassan et al. 2021; Rizk-Allah and El-Fergany 2021; Ismaeel et al. 2021).

##### Initialization

In the initial stage of the optimization process, the GBO generates a random initial population ($P$). The $N \times D$ dimensional population can be formulated as follows (Ahmadianfar et al. 2020):

1 $${P}_{i,d}=\left[{P}_{i,1}, {P}_{i,2},\dots , {P}_{i,D}\right] (i=1,\dots , N) (d=1, 2, \dots , D)$$

2 $${P}_{i}={P}_{lb}+rand\times \left({P}_{ub}-{P}_{lb}\right)$$

where ${P}_{lb}$ and ${P}_{ub}$ show the lower and upper bounds of design parameters. $N$ and $D$ are population size and the count of design parameters, respectively. $rand$ is a randomly selected number in the range of [0, 1].

##### Gradient search rule (GSR)

The GSR is regarded as the most pivotal operator of the GBO algorithm. This rule is based on the concept of regulating the movement of vectors to achieve superior positions within the search space. In this procedure, the initial guess is moved to the next location using a gradient approach, rather than the direct derivation of the function. Consequently, the position of the current vector (${{P}_{i}}^{t}$) is updated by Eq. (3) (Ahmadianfar et al. 2020; Hassan et al. 2021).

3 $${{P1}_{i}}^{t}={{P}_{i}}^{t}-randn\times {\rho }_{1}\times \frac{2 \Delta \text{P}\times {{P}_{i}}^{t}}{({P}_{worst}-{P}_{best}+\varepsilon )}+rand\times {\rho }_{2}\left({P}_{best}-{{P}_{i}}^{t}\right)$$

where ${P}_{best}$ and ${P}_{worst}$ show the best and worst solutions determined based on the fitness value. $randn$ is a random number obtained by the normal distribution. $\varepsilon$ takes a numeric value between 0 and 0.01. ${\rho }_{1}$ is a coefficient used to adjust the balance of exploration and exploitation dynamically. ${\rho }_{2}$ is a random parameter that aims to enhance the exploration ability. $\Delta P$ represents the difference between the ${P}_{best}$ and a randomly selected solution vector (${{P}_{k1}}^{t}$). $\Delta P$ changes are checked at each iteration using Eqs. (4) and (5).

4 $$\Delta P=rand \;\left(1:N\right)\times \left|\frac{\left({P}_{best-}{{P}_{k1}}^{t}\right)+\delta }{2}\right|$$

5 $$\delta =2\times rand\times \left(\left|\frac{{{P}_{k1}}^{t}+{{P}_{k2}}^{t}+{{P}_{k3}}^{t}+{{P}_{k4}}^{t}}{4}-{{P}_{i}}^{t}\right|\right)$$

where $k1$, $k2$, $k3$, and $k4$ ($k1\ne k2\ne k3\ne k4$) are integer numbers randomly selected from the interval of [1, *N*]. A new solution vector (${{P2}_{i}}^{t}$) is obtained by replacing the current vector (${{P}_{i}}^{t}$) used in Eq. (3) with the best vector ${(P}_{best})$.

6 $${{P2}_{i}}^{t}={P}_{best}-randn\times {\rho }_{1}\times \frac{2 \Delta \text{P}\times {{P}_{i}}^{t}}{({{yp}_{i}}^{t}-{{yq}_{i}}^{t}+\varepsilon )}+rand\times {\rho }_{2} \left({{P}_{k1}}^{t}-{{P}_{k2}}^{t}\right)$$

7 $${yp}_{i}=rand \left(\frac{{z}_{i+1}+{P}_{i}}{2}+rand\; \Delta \text{P}\right)$$

8 $${yq}_{i}=rand \left(\frac{{z}_{i+1}+{P}_{i}}{2}-rand\; \Delta \text{P}\right)$$

GBO uses search methods given in Eqs. (3) and (6) exhibit powerful exploration and exploitation capabilities throughout the metaheuristic search process. Finally, the new solution is updated as follows:

9 $${{P}_{i}}^{t+1}={r}_{a}\times ({r}_{b}\times {{P1}_{i}}^{t}+\left(1-{r}_{b}\right)\times {{P2}_{i}}^{t})+\left(1-{r}_{a}\right)\times {{P3}_{i}}^{t}$$

10 $${{P3}_{i}}^{t}={{P}_{i}}^{t}-{\rho }_{1 }\times ({{P2}_{i}}^{t}-{{P1}_{i}}^{t})$$

##### Local escape operator (LEO)

The LEO operator aims to increase GBO’s search performance in order to cope with complex problems. The solution ${{P}_{LEO}}^{t}$ is produced as shown in Algorithm 1 (Rizk-Allah and El-Fergany 2021).


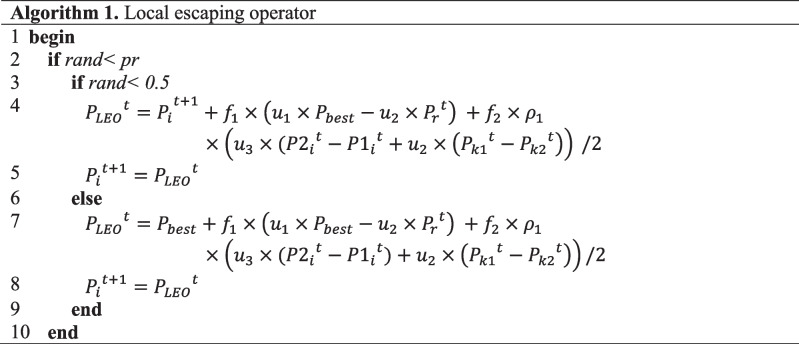


In Algorithm 1, *pr* indicates probability, ${{P}_{r}}^{t}$ depicts a new randomly generated solution, ${f}_{1}$ is a random number in the range of [− 1, 1], and ${f}_{2}$ is a random number produced from a normal distribution (mean 0 and standard deviation 1). ${u}_{1}$, ${u}_{2}$ and ${u}_{3}$ represent random numbers and are defined as shown in Eqs. (11–13).

11 $$u_1=\left\{\begin{array}{cl}2\times rand&if\;\mu_1<0.5\\1&otherwise\end{array}\right.$$

12 $$u_2=\left\{\begin{array}{cl}rand&if\;\mu_1<0.5\\1&otherwise\end{array}\right.$$

13 $$u_3=\left\{\begin{array}{cl}rand&if\;\mu_1<0.5\\1&otherwise\end{array}\right.$$

where $rand$ and ${\mu }_{1}$ are numbers in the range of [0, 1]. ${u}_{1}$, ${u}_{2}$, and ${u}_{3}$ can be simplified as follows:

14 $${u}_{1}={L}_{1}\times 2\times rand+(1-{L}_{1})$$

15 $${u}_{2}={L}_{1}\times rand+(1-{L}_{1})$$

16 $${u}_{3}={L}_{1}\times rand+(1-{L}_{1})$$

where ${L}_{1}$ is a binary number assigned a value of 0 or 1. The value of ${L}_{1}$ is directly associated with ${\mu }_{1}$. If ${\mu }_{1}$ is less than 0.5, ${L}_{1}$ is equal to 1. Otherwise, the value is 0. The solution ${{P}_{r}}^{t}$ is updated according to the scheme given in Eq. (17).

17 $$P_r^t=\left\{\begin{array}{cl}P_{rnd}&if\;\mu_2<0.5\\P_p^t&otherwise\end{array}\right.$$

where ${\mu }_{2}$ is a random number between 0 and 1, and ${P}_{rnd}$ shows a new solution generated using Eq. (2). ${{P}_{p}}^{t}$ is the randomly selected solution from the population. Equation (17) can be written more simply as follows:

18 $${{P}_{r}}^{t}={L}_{2}\times {{P}_{p}}^{t} +\left(1-{L}_{2}\right)\times {P}_{rnd}$$

where ${L}_{2}$ takes a binary value (0 or 1). If ${\mu }_{2}$ is less than 0.5, the value of ${L}_{2}$ is 1; otherwise, it is 0. Random behaviour in the selection of parameters contributes to avoiding local optimum solution traps and increasing population diversity. The optimization framework of the GBO algorithm is depicted in Fig. 2 (Ismaeel et al. 2021; Ahmadianfar et al. 2020).

**Fig. 2:**
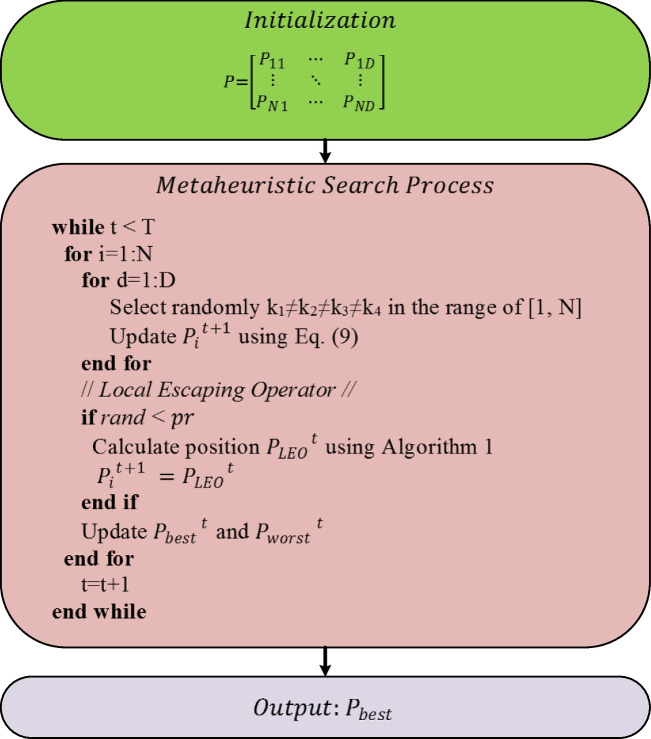
Flowchart of GBO algorithm

#### Harris Hawks optimization (HHO)

HHO is a swarm-based algorithm inspired by the collaborative information sharing observed in Harris Hawks during hunting prey (Heidari et al. 2019). The optimization process of the algorithm can be divided into two stages: the seeking of prey and the hunting of prey. In the initial stage, the members of the population embark on exploratory flights. In the subsequent stage, hunting is conducted by utilizing a range of attack strategies (Kamboj et al. 2020; Qu et al. 2020; Zhang et al. 2020).

##### Initialization

As with other swarm-based metaheuristics, the optimization process in HHO commences with the generation of the initial population. The initial population in the HHO algorithm is generated by Eq. (1).

##### Seeking prey stage

At this stage, the algorithm fulfils the exploration task. To this end, HHO applies two equally probable position update strategies to identify promising solution candidates in the search space. The mathematical model of these strategies is given in Eq. (19) (Heidari et al 2019).

19 $$P\left(t+1\right)=\left\{\begin{array}{ll}P_{rand}\left(t\right)-a_1\left|P_{rand}\left(t\right)-2a_2P\left(t\right)\right|&q\geq0.5\\\left(P_{rabbit}\left(t\right)-P_m\left(t\right)\right)-a_3\left(lb+a_4\left(ub-lb\right)\right)&q<0.5\end{array}\right.$$

where $P\left(t\right)$ and $P\left(t+1\right)$ show position vectors of Harris Hawks for the current and next iteration, respectively. ${P}_{rand}\left(t\right)$ is a randomly selected solution from the population. ${P}_{rabbit}\left(t\right)$ is the position of the rabbit. ${a}_{1}$, ${a}_{2}$, ${a}_{3}$, and ${a}_{4}$ are randomly selected numbers in the range of [0-1]. ${P}_{m}\left(t\right)$ indicate the average position of hawks and defined as follows:

20 $${P}_{m}(t)=\frac{1}{N}\sum\limits_{k=1}^{N}{P}_{i}(t)$$

In the first iterations of the optimization process, hawks search for rabbits. But over time, it is necessary to give importance to catching the rabbit rather than searching for it. This process represents the transition from diversification to intensification. For this transition, the term $E$, which indicates the escape energy of the rabbit, is used (Qu et al. 2020).

21 $$E=2\;{E}_{0} (1-\frac{t}{T})$$

where *t* is the current iteration index and $T$ indicates the maximum number of iterations. ${E}_{0}$ shows the initial energy level and its value is changed in the interval of [− 1, 1] at every iteration. A status of |*E*|≥ 1 shows that the algorithm should focus on exploration, and |*E*|< 1 indicates that it should focus on exploitation.

##### Hunting stage

Once the Harris Hawks identify the prey location, they attack it using one of four pounce strategies: soft besiege, hard besiege, soft besiege with progressive rapid dives, and hard besiege with progressive rapid dives. On the other hand, prey tends to get rid of potential threats. Assuming that *r* represents the probability of prey escaping, *r* >  = 0.5 shows that the prey could not escape, while *r* < 0.5 indicates that the prey could escape. The numerical value of *r* and *E* plays an important role in determining the attack strategy (Heidari et al 2019; Qu et al. 2020).

##### Soft besiege

The soft besiege strategy is applied when |*E*|≥ 0.5 and *r* ≥ 0.5. The rabbit still has high energy to escape. Although the rabbit makes misleading leaps, it eventually fails. Meanwhile, Harris Hawks softly surround their prey to further tire them out. The present attack strategy is modelled as follows:

22 $$P\left(t+1\right)=\Delta P\left(t\right)-E\left|K\times {P}_{rabbit}\left(t\right)-P(t)\right|$$

23 $$\Delta P\left(t\right)={P}_{rabbit}\left(t\right)-P(t)$$

where $K=2 (1-{a}_{5})$ represents the rabbit’s random jump strength. ${a}_{5}$ takes a random value between 0 and 1.

##### Hard besiege

If |*E*|< 0.5 and *r* ≥ 0.5, rabbit has low energy. In this case, hard besiege strategy is applied. The present strategy is modeled as follows:

24 $$P\left(t+1\right)={P}_{rabbit}\left(t\right)-E\left|\Delta P\left(t\right)\right|$$

##### Soft besiege with progressive rapid dives

When *r* > 0.5 and *E* > 0.5, hawks perform soft besiege with progressive rapid dives. This attack strategy is modelled as follows:

25 $$Y={P}_{rabbit}\left(t\right)-E\left|K\times {P}_{rabbit}\left(t\right)-P(t)\right|$$

where $Y$ indicates the next move that the Hawks obtain based on soft besiege. If it is determined that the prey is making deceptive movements, sudden and fast dives are made using the Levy flight concept as defined in Eqs. (26–28).

26 $$Z=Y+S \times \mathrm{Levy\; Flight }\;(\text{dim})$$

27 $$\text{Levy Flight}=0.01\times \frac{u\times \sigma }{{\left|v\right|}^{1/\beta }}$$

28 $$\sigma ={\left\{\frac{\Gamma \left(1+\beta \right)\times \text{sin}(\frac{\pi \beta }{2})}{\Gamma \left[\frac{1+\beta }{2}\right]\times \beta \times {2}^{((\beta -1)/2)} }\right\}}^{1/\beta }$$

where $S$ is a random vector by 1 $\times$ D dimensional. $u$ and $v$ show randomly selected numbers in the interval of (0–1). $\Gamma$ is gamma distribution. $\beta$ is a numeric number with a value equal to 1.5. Finally, the location is updated according to Eq. (29). In that equation, $f$ illustrates the fitness function.

29 $$P\left(t+1\right)=\left\{\begin{array}{cc}Y&if\;f\left(Y\right)<f(P(t))\\Z&if\;f\left(Z\right)<f(P(t))\end{array}\right.$$

##### Hard besiege with progressive rapid dives

In the case of |*E*|< 0.5 and *r* < 0.5, the prey (rabbit) has limited energy to escape. This indicates that Harris Hawks can catch their prey without difficulty. In this case, the hard besiege with progressive rapid dives strategy comes into play. This strategy narrows the siege by reducing the distance between the Harris Hawks and the prey to avoid potential loss of prey. Mathematically, this strategy can be formulated as follows:

30 $$P\left(t+1\right)=\left\{\begin{array}{cc}Y&if\;f\left(Y\right)<f(P(t))\\Z&if\;f\left(Z\right)<f(P(t))\end{array}\right.$$

*Y* and *Z* are calculated as shown in Eqs. (31) and (32), respectively.

31 $$Y={P}_{rabbit}\left(t\right)-E\left|K\times {P}_{rabbit}\left(t\right)-{P}_{m}\left(t\right)\right|$$

32 $$Z=Y+S \times \mathrm{Levy\; Flight }\;(dim)$$

**Fig. 3:**
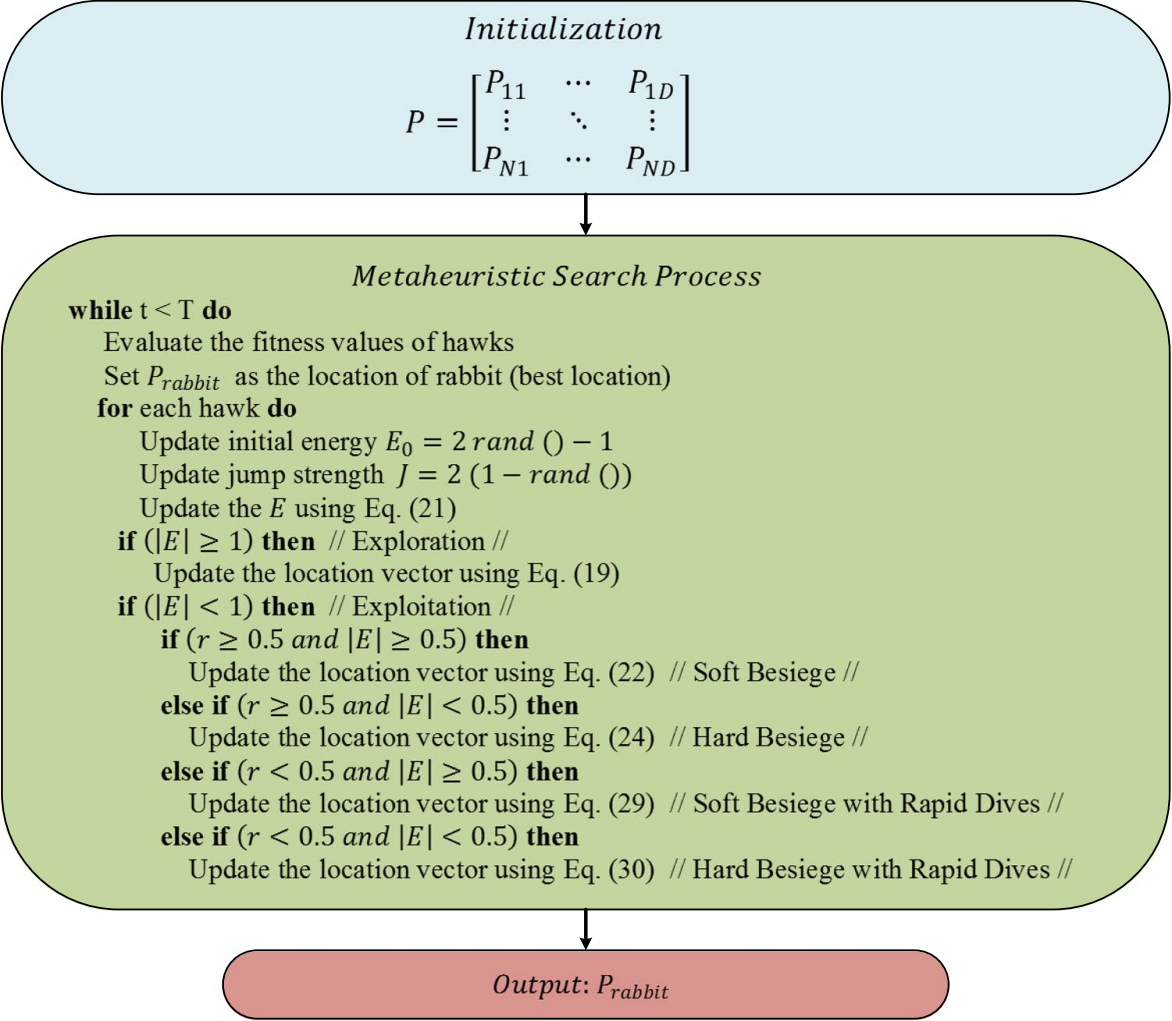
Flowchart of HHO algorithm

Figure 3 illustrates the optimization framework of the HHO algorithm (Heidari et al 2019).

#### Barnacles mating optimizer (BMO)

BMO is an evolutionary metaheuristic that has been developed by mimicking the copulation conduct of barnacles (Sulaiman et al. 2020). The optimization framework of the algorithm is introduced as follows:

##### Initialization

In the BMO algorithm, the search agent is named Barnacles. The population vector ($P$) containing the search agents is created using Eqs. (1) and (2).

##### Selection process

Unlike other evolutionary algorithms, the BMO considers penis length (*pl*) when choosing barnacles to pair. The selection process is made considering various assumptions. Detailed information on these assumptions is given in Sulaiman et al. (2020). The search agents are selected using the following equations (Sulaiman et al. 2020; Agwa et al. 2022):

33 $$barnacle\_d=randperm(N)$$

34 $$barnacle\_m=randperm(N)$$

where $barnacle\_d$ and $barnacle\_m$ show the parents to be mated. $N$ is the population size.

##### Reproduction

The penis length (*pl*) is of great importance in managing the exploration and exploitation process. If the selection of barnacles to be mated is equal to penis length, offspring generation is performed with Eq. (35) (Sulaiman and Mustaffa 2021):

35 $${{P}_{i}}^{D\_new}=\delta {{P}^{D}}_{barnacle\_d}+\beta {{P}^{D}}_{barnacle\_m}$$

where ${{P}^{D}}_{barnacle\_d}$ and ${{P}^{D}}_{barnacle\_m}$ represent the variables of the father and mother barnacles selected using Eqs. (33) and (34). $\delta$ and $\beta$ indicate the percentage of characteristics of the father and mother that are transmitted to the offspring. For instance, let us assume that $\delta$ is 0.65, which means that 65% of the Father’s solutions and 35% of the Mother’s solutions are embedded in the new offspring solution.

**Fig. 4:**
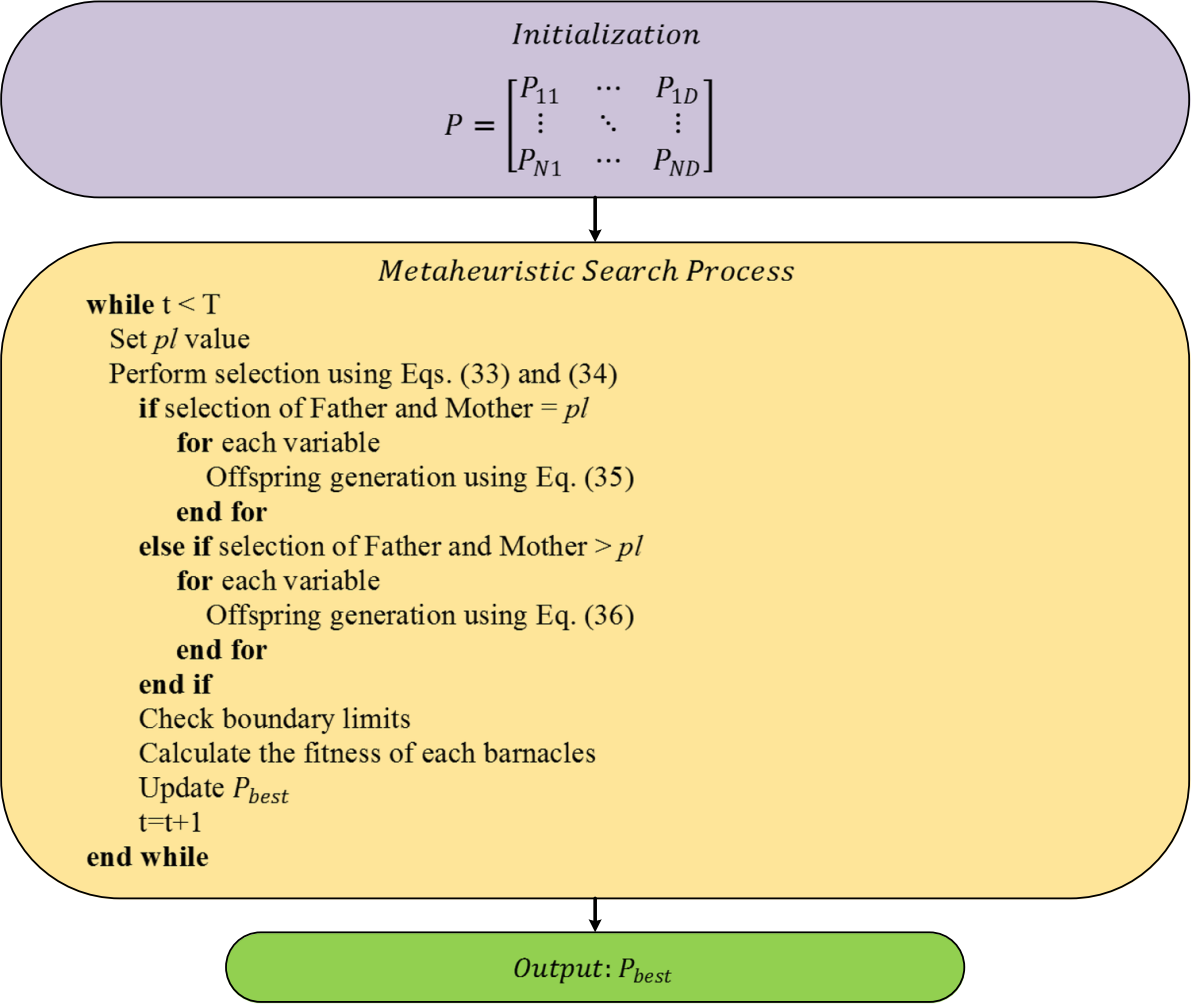
Flowchart of BMO algorithm

If the selection of barnacles to be mated exceeds the predetermined *pl* value, offspring generation is performed with Eq. (36). The optimization scheme of the BMO algorithm is given in Fig. 4 (Sulaiman et al. 2020; Ahmed et al. 2020).

36 $${{P}_{i}}^{N\_new}=rand ()\times {{P}^{N}}_{barnacle\_m}$$

#### Sine cosine algorithm (SCA)

SCA is a population-based optimizer inspired by the mathematical model of sine and cosine functions (Mirjalili 2016). As with other metaheuristics, the optimization process of SCA starts with the generating of the random initial population as shown in Eq. (1). Then it moves on to the metaheuristic search process where exploitation and exploration tasks are fulfilled. In this process, the algorithm uses adaptive and random variables to find promising high-quality solutions and intensify the search around these solutions. Equations (37) and (38) are applied to reach these targets (Sarwagya et al. 2020; Bhookya and Jatoth 2019; Das et al. 2018).

37 $${{P}_{i,j}}^{t+1}={{P}_{i,j}}^{t}+{m}_{1}\times \text{ sin}({m}_{2})\times \left|{m}_{3} {{TL}_{1,j}}^{t}-{{P}_{i,j}}^{t}\right|$$

38 $${{P}_{i,j}}^{t+1}= {{P}_{i,j}}^{t}+{m}_{1}\times \text{ cos}({m}_{2})\times \left|{m}_{3} {{TL}_{1,j}}^{t}-{{P}_{i,j}}^{t}\right|$$

where $i=1,\dots , N$ and $j=1, 2, \dots , D$. ${{P}_{i,j}}^{t}$ shows the location of search agent in *j*-th dimension at iteration *t*. ${{TL}_{1,j}}^{t}$ is the location of destination point in *j*-th dimension. ${m}_{1}$, ${m}_{2}$, and ${m}_{3}$ are the random numbers. The above equations can be formulated as a single equation based on a condition as shown in Eq. (39) (Mirjalili 2016).

39 $${{P}_{i,j}}^{t+1}= \left\{\begin{array}{c}{{P}_{i,j}}^{t}+{m}_{1}\times \text{ sin}({m}_{2})\times \left|{m}_{3} {{TL}_{1,j}}^{t}-{{P}_{i,j}}^{t}\right|, {m}_{4}<0.5\\ {{P}_{i,j}}^{t}+{m}_{1}\times \text{ cos}({m}_{2})\times \left|{m}_{3} {{TL}_{1,j}}^{t}-{{P}_{i,j}}^{t}\right|, {m}_{4}\ge 0.5\end{array}\right.$$

**Fig. 5:**
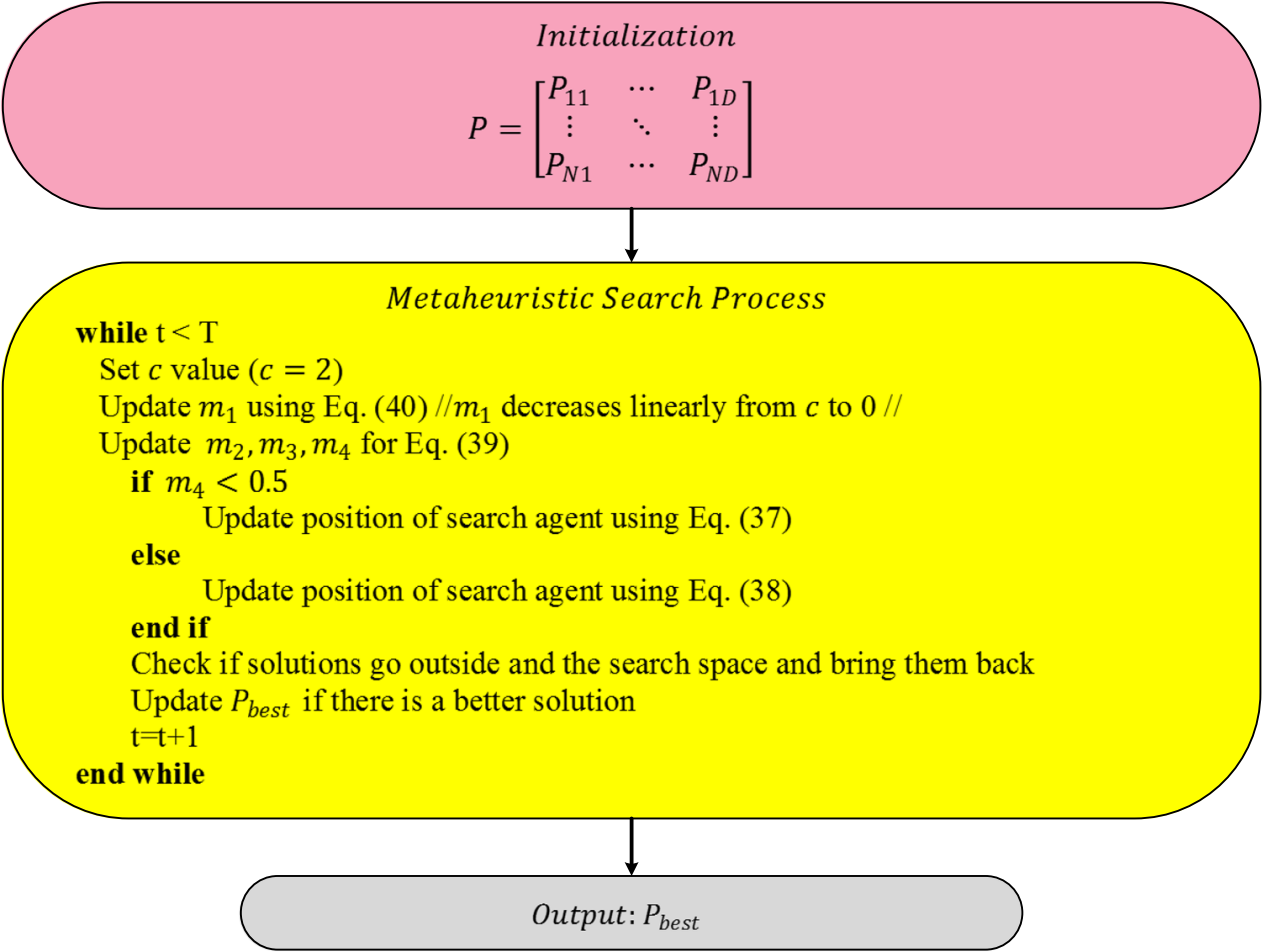
Flowchart of SCA algorithm

SCA uses an adaptive parameter to balance exploration and exploitation. To achieve this target, ${m}_{1}$ is updated over iterations using Eq. (40). In that equation, $c$ represents a constant number. The optimization framework of SCA is given in Fig. 5 (Mirjalili 2016).

40 $${m}_{1}=c- t \left(\frac{c}{T}\right)$$

#### Henry gas solubility optimization (HGSO)

HGSO is a physics-based metaheuristic algorithm inspired by Henry’s law (Hashim et al. 2019). Henry’s law describes the phenomenon related to the solubility of a gas in a liquid at a given pressure. The algorithm mimics the huddling behaviour of gas in the process of finding the optimal values (Neggaz et al. 2020; Mirza et al. 2020). The mathematical model of HGSO is introduced in detail below.

##### Initialization

The population ($P$) representing the gas particles to be dissolved in a given liquid is formed as shown in Eqs. (1) and (2). Each solution candidate (${P}_{i}$) is called a gas. The properties of each gas including Henry’s constant of type *j* (${H}_{j}$), partial pressure of *i*-th gas in cluster *j* (${Zr}_{i,j}$), and ${\nabla }_{sol}E/R$ constant value of type *j* (${C}_{j}$) are defined as follows (Hashim et al. 2019):

41 $$Gas\;properties=\left\{\begin{array}{c}H_j\left(t\right)=l_1\times rand\;(0,1)\\{Zr}_{i,j}=l_2\times rand\;(0,1)\\C_j=l_3\times rand\;(0,1)\end{array}\right.$$

where ${l}_{1}$, ${l}_{2}$, and ${l}_{3}$ are constant numbers. Their values are 0.05, 100, and 0.01, respectively.

##### Clustering

Depending on the number of gas species, the population is split into equal clusters. It is assumed that each cluster has homogeneous gases and thus uses the same Henry’s constant value (${H}_{j}$) (Neggaz et al. 2020).

##### Evaluation

The evaluation step is based on the consideration of checking the best solubility of the gases. Firstly, the gas with the best fitness value is determined in each cluster (i.e., ${P}_{j,best}$). Then, the whole population is ranked and the global best (${P}_{best}$) of the population is selected (Hashim et al. 2019).

##### Update Henry’s coefficient

The Henry coefficient is updated over the iterations using the formula given in Eq. (42) (Neggaz et al. 2020).

42 $${H}_{j}\left(t+1\right)= {H}_{j}\left(t\right)\times \text{exp}\left(-{C}_{j}\times \left(\frac{1}{\text{exp}(-t/T)} -\frac{1}{{T}^{Q}}\right) \right)$$

where ${H}_{j}$ shows the Henry’s coefficient for cluster *j*. ${T}^{Q}$ is the constant number and its value is 298.15.

##### Update solubility

The value of gas solubility is updated using the formula as given in Eq. (43). In that equation, ${S}_{i,j}$ depicts the solubility of *i-*th gas in cluster *j*, $k$ is a constant number, and ${Pr}_{i,j}$ indicates the pressure on *i-*th gas in cluster *j* (Hashim et al. 2019)*.*

43 $${S}_{i,j}\left(t\right)= k\times {H}_{j}\left(t+1\right)\times {Zr}_{i,j}\left(t\right)$$

##### Update position

The position of gas *i* in cluster *j* is updated as follows (Hashim et al. 2019):

44 $${P}_{i,j}\left(t+1\right)= {P}_{i,j}\left(t\right)+F\times r\times \vartheta \times \left({P}_{i,best}\left(t\right)-{P}_{i,j}\left(t\right)\right)+F\times r\times \varphi \times ({S}_{i,j}\left(t\right)\times {P}_{best}\left(t\right)-{P}_{i,j}\left(t\right))$$

45 $$\vartheta =\Phi \times \text{exp}\times \left(-\frac{{f}_{best}\left(t\right)+\varepsilon }{{f}_{i, j}\left(t\right)+\varepsilon }\right), \varepsilon =0.05$$

where ${P}_{i,j}$ indicates the position of *i*-th gas in cluster *j*. $r$ is the random constant. $F$ parameter gives information about the change of the search agent direction. ${P}_{i,best}\left(t\right)$ is the best gas *i* in cluster *j*. ${P}_{best}$ depicts the best gas in population. $\vartheta$ is defined as the gas’s ability to interact with other gases in the cluster. $\varphi$ is the influence of other gases on the *i-*th gas in cluster *j*. $\Phi$ is a constant value. ${f}_{i, j}$ represents the fitness of *i*-th gas in cluster *j*.

##### Escape from local optimum

This step contributes to eliminating the premature convergence problem of the HGSO. In this direction, the ranking and selection of the worst solutions (${N}_{w}$) are performed using Eq. (46) (Neggaz et al. 2020).

46 $${N}_{w}= N\times \left(rand \left({c}_{2}-{c}_{1}\right)+{c}_{1}\right) , {c}_{1}=0.1\text{ and }{c}_{2}=0.2$$

##### Update position of the worst agents

**Fig. 6:**
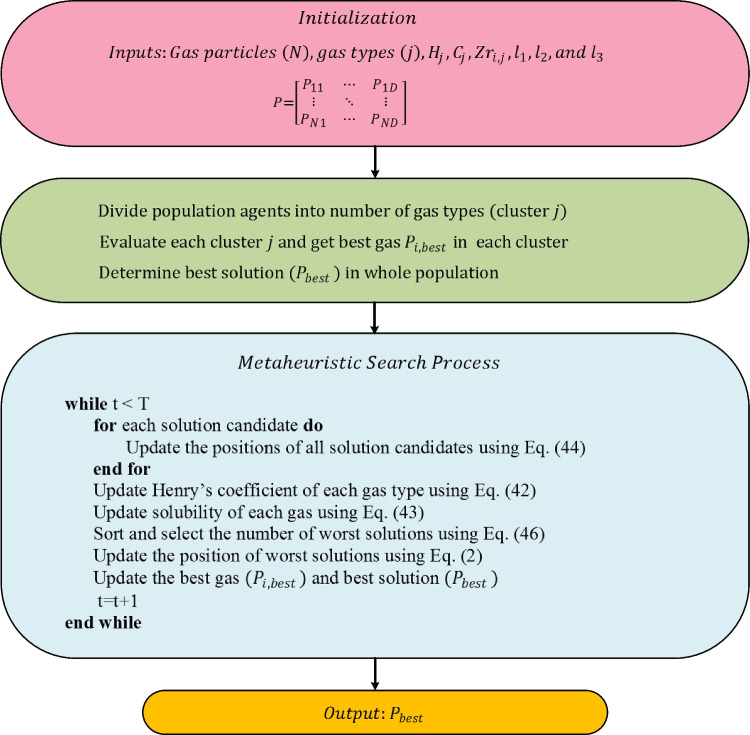
Flowchart of HGSO algorithm

The position of the worst agents is updated using Eq. (2). The optimization framework of the HGSO algorithm is given in Fig. 6 (Hashim et al. 2019).

### Evaluation benchmarks

Prediction accuracy is an important indicator in evaluating algorithm performance. The error metrics are commonly used both to evaluate the prediction results and to demonstrate the algorithm’s performance. In the present study, the prediction performance of algorithms is evaluated by the coefficient of determination (*R*^2^), mean absolute bias error (MABE), root mean squared error (RMSE), and mean bias error (MBE) statistical metrics. Table 2 gives the definitions of the statistical performance metrics. In Table 2, the ${G}^{measured}$ and ${G}^{predicted}$ are the measured and predicted GSR data, respectively. The number of observations and the mean value of the measured GSR data are represented by $M$ and $\overline{{{G }_{m}}^{measured}}$, respectively.

**Table 2:** Definitions of statistical metrics

Metrics	Mathematical model	Definition
*R*^2^	$1-\frac{\sum {({{G}_{m}}^{measured}-{{G}_{m}}^{predicted})}^{2}}{\sum {({{G}_{m}}^{measured}-\overline{{{G }_{m}}^{measured}})}^{2}}$	The determination coefficient (*R*^2^) is a measure of how well a model can predict a set of actual data. The relevant metric takes values in the range of 0 to 1, and a value close to 1 means better performance (Gouda et al. 2019)
MABE	$\frac{1}{M} \sum\limits_{m=1}^{M}\left|{{G}_{m}}^{predicted}-{{G}_{m}}^{measured}\right|$	MABE measures the absolute value of the bias error. A value close to zero indicates a better correlation between the predicted and measured data. It reflects the long-term performance of prediction models (Yang et al. 2020)
RMSE	$\sqrt{\frac{1}{M}\sum\limits_{m=1}^{M}{({{G}_{m}}^{predicted}-{{G}_{m}}^{measured})}^{2}}$	RMSE is the standard deviation of the prediction errors. It always takes a positive value and is desired to be close to zero. It is a significant metric for the short-term performance of the prediction method (Zang et al. 2020)
MBE	$\frac{1}{M} \sum\limits_{m=1}^{M}({{G}_{m}}^{predicted}-{{G}_{m}}^{measured})$	MBE shows whether the prediction method overestimates or underestimates the actual data. A small MBE value indicates better performance of the prediction method (Fan et al. 2018a)

### Formulation of global solar radiation prediction problem

In this paper, the prediction of daily GSR data is modelled by using the linear form as given in Eq. (47) (Kıran et al. 2012). The input parameters of the linear model are sunshine duration (${X}_{1}$), actual pressure (${X}_{2}$), moisture ${(X}_{3})$, wind speed (${X}_{4}$), and ambient temperature (${X}_{5}$), respectively. The weight coefficients (${w}_{1}\dots {w}_{6}$) are tuned by the GBO, HHO, BMO, SCA, and HGSO algorithms with minimization of the objective function given in Eq. (48) (Tefek et al. 2019).

47 $${G}^{predicted}={w}_{1}{ X}_{1}+ {w}_{2}{ X}_{2}+{w}_{3}{ X}_{3}+{w}_{4}{ X}_{4}+{w}_{5}{ X}_{5}+{w}_{6}$$

48 $$\text{min}\;F(v)= \sqrt{ \frac{1}{M} \sum\limits_{m=1}^{M}{({{G}_{m}}^{predicted}-{{G}_{m}}^{measured})}^{2}}$$

In the study, all algorithms were coded using MATLAB® R2023b software. The simulation is performed on a machine with the following specifications: Intel Core i5-3210 M @ 2.50 GHz, 6 GB RAM, and an × 64-based processor. The maximum number of iterations (*T*) is set to 2000 for all algorithms. The algorithms were run with the parameter settings specified in the original articles.

**Fig. 7:**
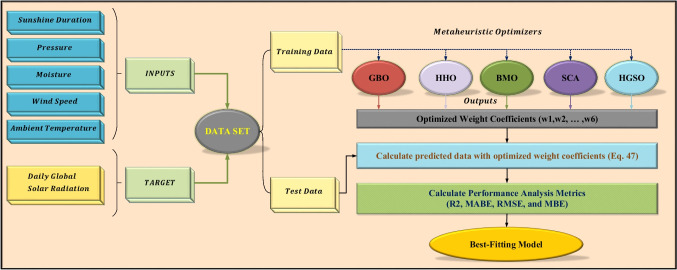
Flowchart of daily global solar radiation prediction with metaheuristic optimizers

Figure 7 presents a pictorial representation of the daily global solar radiation prediction using metaheuristic algorithms. As shown in the figure, the dataset includes historical data on sunshine duration, actual pressure, moisture, wind speed, ambient temperature, and daily global solar radiation. The dataset, which encompasses data from 1 January 2010 to 31 December 2018, has been divided into two distinct sections: training data, which spans the years between 2010 and 2017, and testing data, which encompasses the year 2018. Metaheuristic algorithms are calibrated using the training data and then employed to identify the optimal settings for the weight coefficients. The predicted GSR data for the test data is calculated by substituting the optimized weight coefficients into Eq. (47). Subsequently, the efficacy of the algorithms is evaluated through the application of statistical evaluation metrics, namely *R*^2^, MABE, RMSE, and MBE, which quantify the fitting between the predicted and actual data.

## Results and discussion

This paper aims to estimate the daily solar radiation of three provinces in Türkiye, namely Afyonkarahisar, Rize and Ağrı. In this direction, GBO, HHO, BMO, SCA, and HGSO algorithms are used. The algorithms are trained with five input parameters. These are sunshine duration (hours), actual pressure (hPa), moisture (%), wind speed (m/s), and ambient temperature (°C). The dataset covering the years 2010 and 2018 is divided into two categories. The daily data between 2010 and 2017 years is used for training metaheuristic algorithms, while the remaining data (in 2018 year) is utilized to test the performance of the algorithms. The optimized weight coefficients with GBO, HHO, BMO, SCA, and HGSO algorithms are given in Table 3. To evaluate the prediction accuracy of the algorithms, *R*^2^, MABE, RMSE, and MBE statistical benchmark metrics are used. The statistical metric values calculated for the testing phase are reported in Table 4.

**Table 3:** Optimized weight coefficients

Algorithm	Weight coefficients	Afyonkarahisar	Rize	Ağrı
GBO	*w*_1_	0.3546	0.4386	0.3631
	*w*_2_	− 0.0419	− 0.0194	− 0.041
	*w*_3_	− 0.0147	0.0056	− 0.0024
	*w*_4_	0.2275	0.2249	0.0882
	*w*_5_	0.0689	0.0578	0.0627
	*w*_6_	39.249	19.3292	35.7888
HHO	*w*_1_	0.3363	0.4716	0.3496
	*w*_2_	0.0018	0.0007	0.0021
	*w*_3_	− 0.0155	0.0020	− 0.0143
	*w*_4_	0.3054	0.0081	0.3917
	*w*_5_	0.0779	0.0067	0.0343
	*w*_6_	− 0.0727	0.0034	0.4604
BMO	*w*_1_	0.3364	0.4353	0.3433
	*w*_2_	0.0026	− 0.0002	0.0019
	*w*_3_	− 0.0152	0.0037	− 0.0043
	*w*_4_	0.3107	0.2653	0.1515
	*w*_5_	0.0780	0.0686	0.0650
	*w*_6_	− 0.7843	− 0.1456	0.0845
SCA	*w*_1_	0.3510	0.4256	0.3065
	*w*_2_	− 0.0338	0.0004	− 0.0868
	*w*_3_	− 0.0179	− 0.0013	− 0.0197
	*w*_4_	− 0.0185	− 0.0009	− 0.0361
	*w*_5_	0.0842	0.0692	0.0543
	*w*_6_	32.4330	− 0.2226	75.7956
HGSO	*w*_1_	0.3421	0.4517	0.3310
	*w*_2_	0.0002	− 0.00004	0.0018
	*w*_3_	− 0.0211	0.0021	0.0013
	*w*_4_	0.1978	0.0284	0.0123
	*w*_5_	0.0714	0.0646	0.0807
	*w*_6_	1.9079	− 0.0251	0.0352

**Table 4:** Statistical metric results of algorithms for Afyonkarahisar, Rize, and Ağrı provinces

Provinces	Metric	GBO	HHO	BMO	SCA	HGSO
Afyonkarahisar	*R*^2^	**0.8474**	0.8383	0.8380	0.8458	0.8371
	MABE(MJ/m^2^)	0.7188	0.7424	0.7386	**0.7023**	0.7322
	RMSE(MJ/m^2^)	0.9348	0.9583	0.9498	**0.9121**	0.9528
	MBE (MJ/m^2^)	0.2724	0.2802	0.2447	**0.2430**	0.2771
Rize	*R*^2^	**0.8978**	0.8543	0.8949	0.8962	0.8951
	MABE(MJ/m^2^)	0.5895	0.7265	0.5953	0.5913	**0.5767**
	RMSE(MJ/m^2^)	0.7265	0.8564	0.7263	0.7298	**0.7143**
	MBE (MJ/m^2^)	0.0201	0.1425	**0.0140**	0.0477	0.0515
Ağrı	*R*^2^	**0.8810**	0.8517	0.8681	0.8652	0.8677
	MABE(MJ/m^2^)	**0.6703**	0.7359	0.7164	0.7113	0.7389
	RMSE(MJ/m^2^)	**0.8432**	0.9276	0.8845	0.8960	0.9016
	MBE (MJ/m^2^)	0.0451	0.0739	0.0371	**0.0355**	− 0.0810

Bold values show that the best result of the relevant metric

**Fig. 8:**
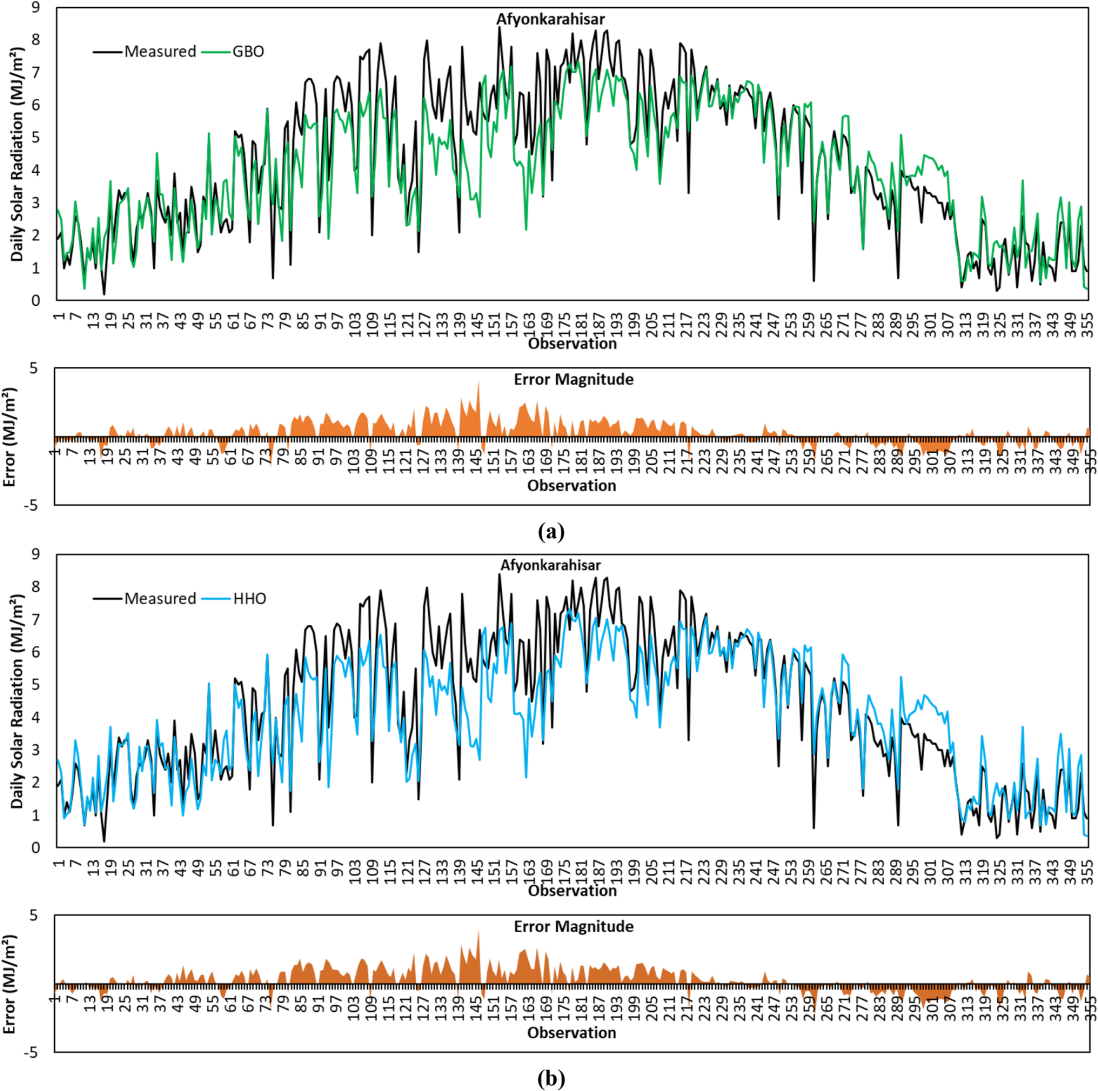

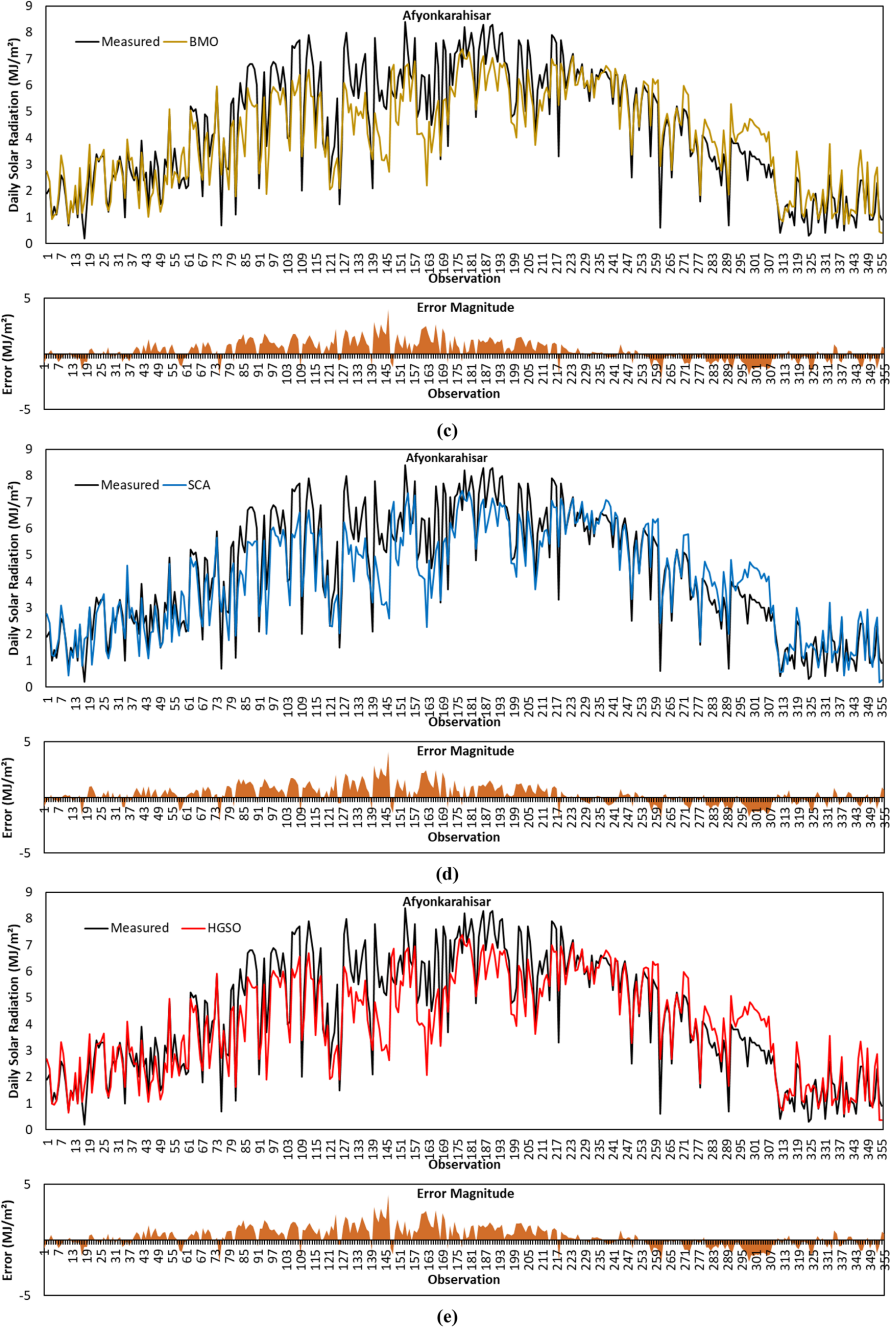
Measured GSR data, forecasting results, and error magnitudes for Afyonkarahisar. **a** GBO. **b** HHO. **c** BMO. **d** SCA. **e** HGSO

Figure 8a depicts predicted GSR data with the GBO algorithm for the Afyonkarahisar province. From the comparative statistical metric results given in Table 4, it is seen that GBO provided the highest *R*^2^ result with a value of 0.8474 for Afyonkarahisar province. Based on MABE and RMSE metric results, GBO obtained the second-best accuracy performance after SCA. GBO’s positive calculation of MBE for Afyonkarahisar means that the average of the solar radiation estimates by the algorithm is lower than the average of the actual data.

The forecasted GSR data by the HHO algorithm for the Afyonkarahisar province is shown in Fig. 8b. As per the results in Table 4, HHO provided better *R*^2^ metric result compared to BMO and HGSO algorithms. The comparison based on MABE, RMSE, and MBE metric results revealed that HHO’s prediction accuracy for Afyonkarahisar province is behind its competitors.

Figure 8c illustrates predicted GSR data by the BMO algorithm for the Afyonkarahisar province. Based on the data given in the figure, the *R*^2^, MABE, RMSE, and MBE metric results of the algorithm are calculated as 0.8380, 0.7386 MJ/m^2^, 0.9498 MJ/m^2^, and 0.2447 MJ/m^2^, respectively. The algorithm presented the second-best MBE metric result.

The forecasted GSR data with the SCA algorithm for the Afyonkarahisar province is shown in Fig. 8d. Considering the statistical metric results given in Table 4, it is observed that the SCA algorithm has achieved the best MABE, RMSE, and MBE results with a value of 0.7023 MJ/m^2^, 0.9121 MJ/m^2^, and 0.2430 MJ/m^2^, respectively. Figure 8d displays that the error magnitudes of SCA are higher in the observations between 76 and 213.

Figure 8e shows the forecasting results obtained by the HGSO algorithm for the Afyonkarahisar province. For the GSR data in the figure, *R*^2^, MABE, RMSE, and MBE metrics are calculated as 0.8371, 0.7322 MJ/m^2^, 0.9528 MJ/m^2^, and 0.2771 MJ/m^2^, respectively. The MABE, RMSE, and MBE metric results of the algorithm are better than HHO. However, the worst *R*^2^ result belongs to the HGSO algorithm.

To summarize the GSR data forecasting results for Afyonkarahisar province, the SCA algorithm achieved the best prediction accuracy with MABE of 0.7023 MJ/m^2^, RMSE of 0.9121 MJ/m^2^, and MBE of 0.2430 MJ/m^2^, respectively. The highest *R*^2^ value is calculated with the GBO algorithm. The algorithm with the weakest prediction accuracy for the same province is HHO.

**Fig. 9:**
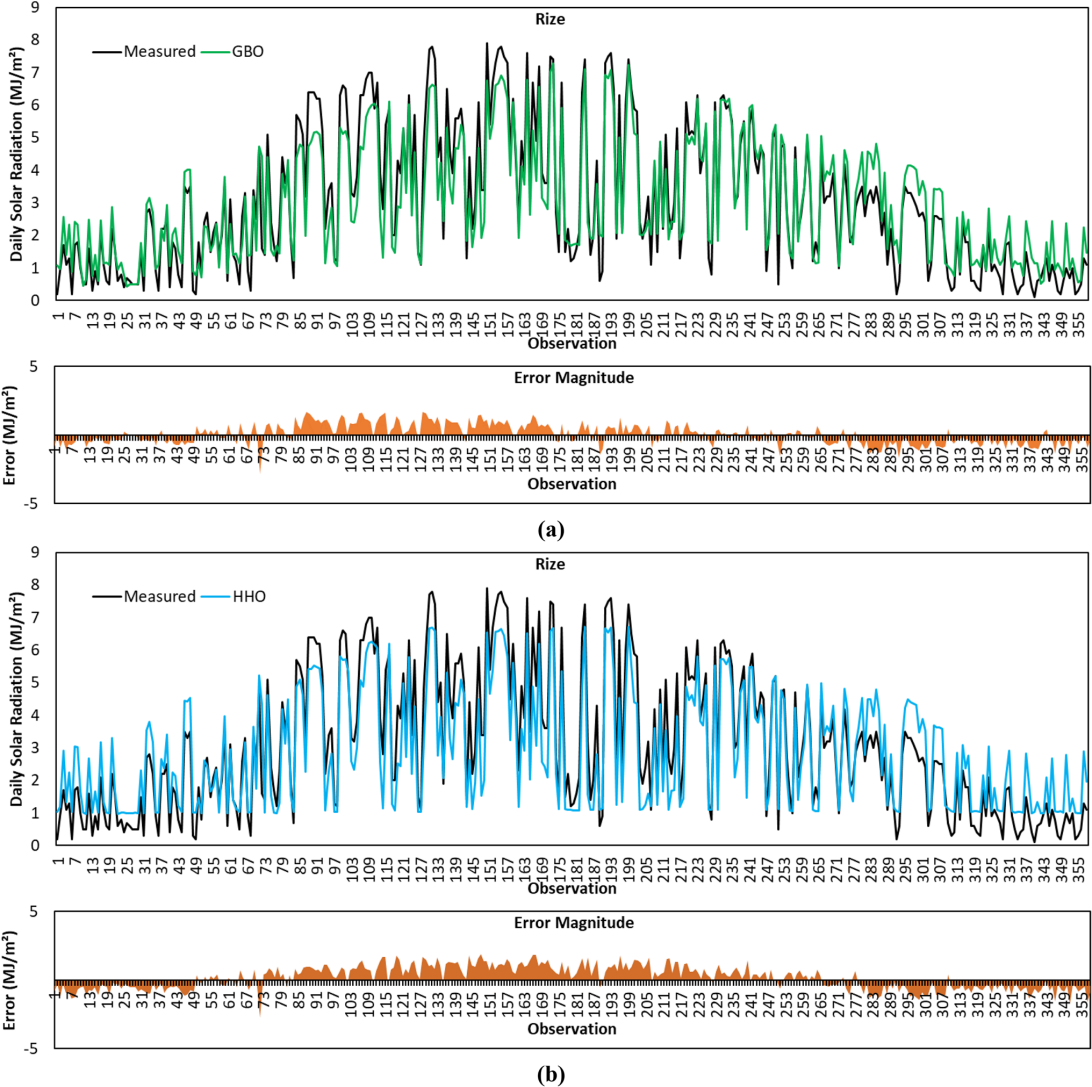

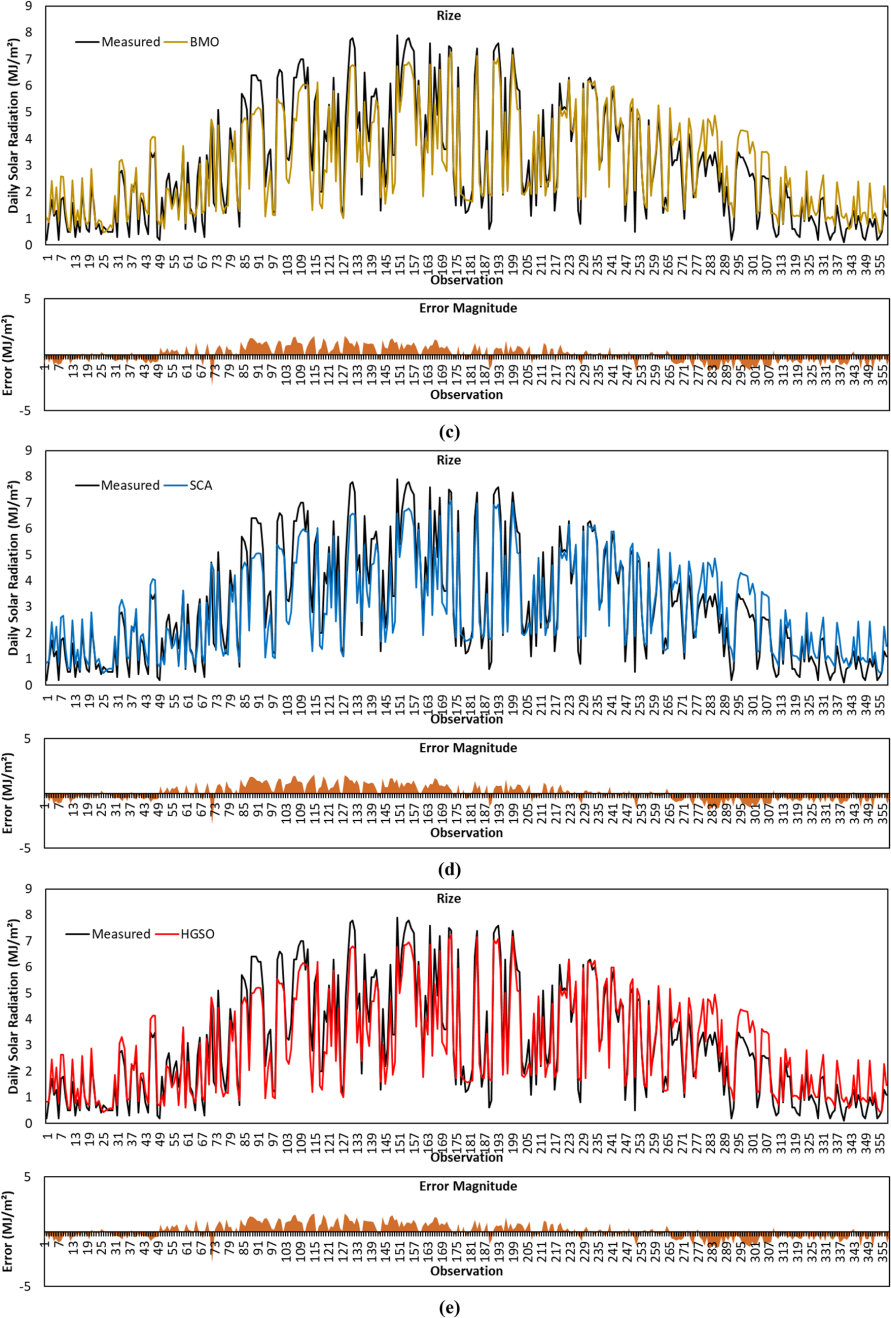
Measured GSR data, forecasting results, and error magnitudes for Rize. **a** GBO. **b** HHO. **c** BMO. **d** SCA. **e** HGSO

Figure 9a shows the GSR data forecasted by the GBO algorithm for Rize province. In this province, *R*^2^ of 0.8978, MABE of 0.5895 MJ/m^2^, RMSE of 0.7265 MJ/m^2^, and MBE of 0.0201 MJ/m^2^ for forecasted GSR data with the GBO algorithm. From the statistical metric results reported in Table 4, it is seen that GBO achieves the highest *R*^2^ result. The results of the remaining metrics showed that the algorithm performed competitively with HGSO and ranked second regarding MABE metric result. As shown in Fig. 9a, the error magnitudes of the GBO algorithm are very low, particularly in the 1–72 and 223–265 observation ranges.

The forecasted GSR data with the HHO algorithm for Rize province is displayed in Fig. 9b. MABE, RMSE, and MBE error metrics between the measured and predicted GSR data given in the figure are calculated as 0.7265 MJ/m^2^, 0.8564 MJ/m^2^, and 0.1425 MJ/m^2^, respectively. Considering all metric results calculated for Rize province, it is observed that HHO gives the worst prediction accuracy among all algorithms.

The plot of predicted GSR data with the BMO algorithm for Rize province is given in Fig. 9c. As revealed in Table 4, the result of *R*^2^, MABE, RMSE, and MBE metrics are calculated to be 0.8949, 0.5953 MJ/m^2^, 0.7263 MJ/m^2^, 0.0140 MJ/m^2^ for BMO algorithm, respectively. The lowest MBE value is obtained by the BMO algorithm for Rize province. The algorithm ranked second among all algorithms based on the RMSE metric.

Figure 9d visualizes the GSR data predicted by the SCA algorithm for Rize province. As can be seen in Table 4, *R*^2^, MABE, RMSE, and MBE results of the SCA algorithm are 0.8962, 0.5913 MJ/m^2^, 0.7298 MJ/m^2^, and 0.0477 MJ/m^2^, respectively. SCA algorithm performed more superbly than HHO, BMO, and HGSO in terms of *R*^2^ value. Considering Fig. 9d, it is seen that the error magnitude of SCA is lower in 1–49 and 223–265 observations.

The predicted GSR data with the HGSO algorithm for Rize province is plotted in Fig. 9e. As per the metric results in Table 4, the results of *R*^2^, MABE, RMSE, and MBE for HGSO algorithm are 0.8951, 0.5767 MJ/m^2^, 0.7143 MJ/m^2^, and 0.0515 MJ/m^2^, respectively. The MABE and RMSE metric values of the algorithm are lower than all algorithms.

Given that all metric results are together for Rize province, it is noticed that the HGSO algorithm provided the most accurate prediction with the lowest MABE (0.5767 MJ/m^2^) and RMSE (0.7143 MJ/m^2^) metrics. For the same province, the highest *R*^2^ (0.8978) and lowest MBE (0.0140 MJ/m^2^) results are achieved by GBO and BMO algorithms, respectively.

**Fig. 10:**
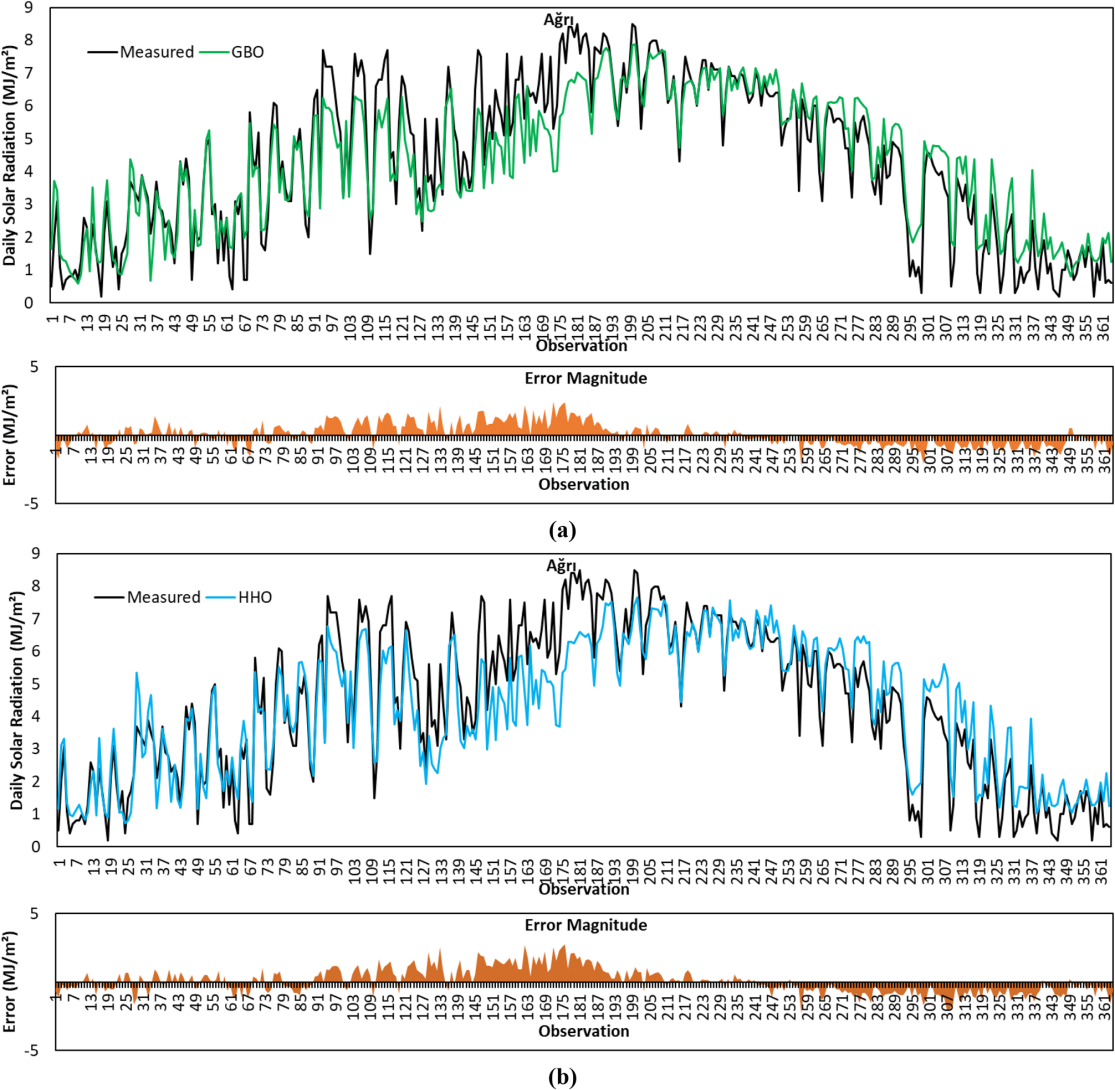

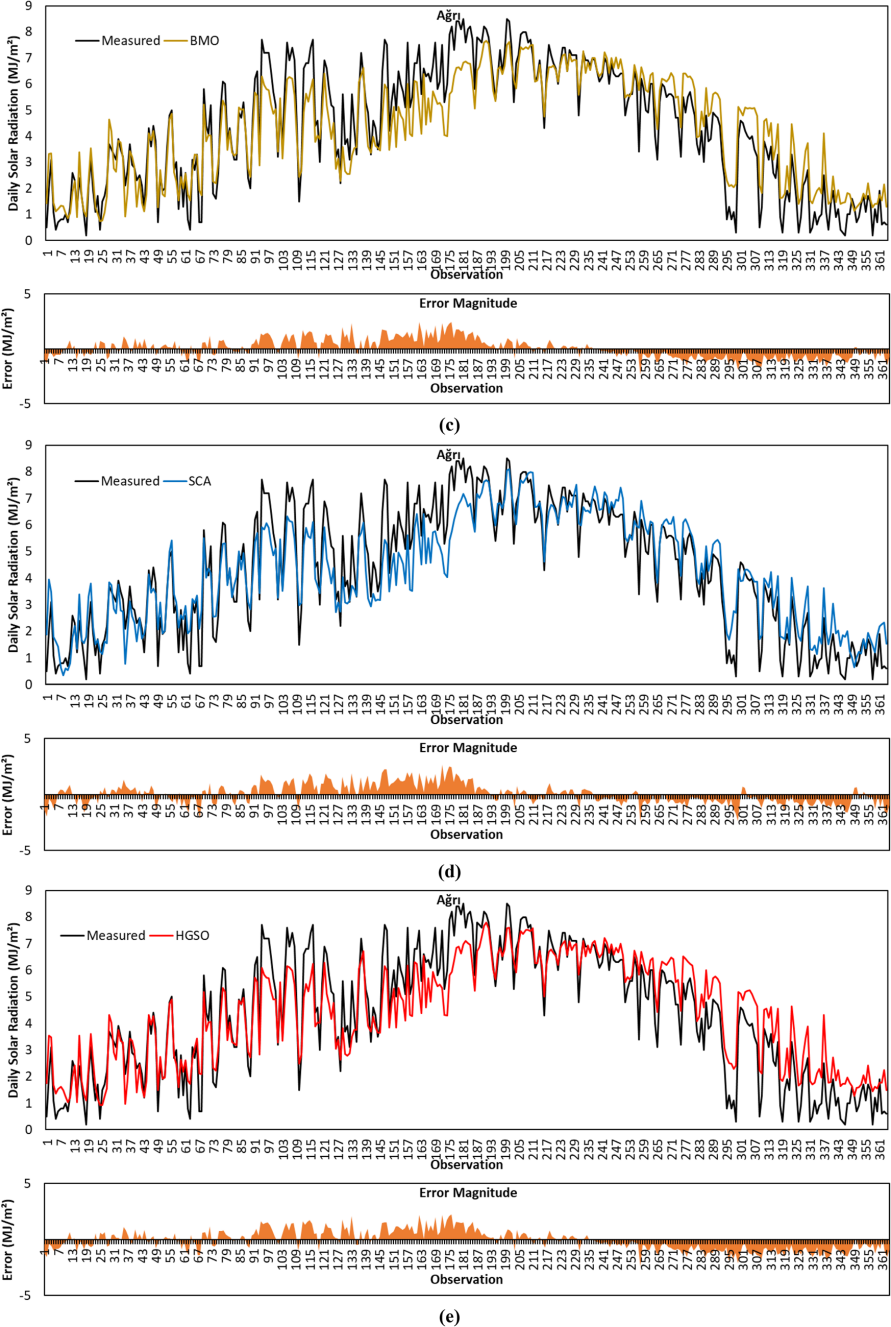
Measured GSR data, forecasting results, and error magnitudes for Ağrı. **a** GBO. **b** HHO. **c** BMO. **d** SCA. **e** HGSO

The forecasted GSR data with the GBO algorithm for Ağrı province is plotted in Fig. 10a. As per the results in Table 4, the algorithm obtained the best results with an *R*^2^ of 0.8810, MABE of 0.6703 MJ/m^2^, and RMSE of 0.8432 MJ/m^2^. Given the results of statistical metrics together, it is seen that the GBO is the best-fitting algorithm in the prediction of GSR data in Ağrı province.

Figure 10b demonstrates the trend of GSR data predicted with the HHO algorithm for the Ağrı province. The MABE, RMSE, and MBE metric results for the data illustrated in the figure are calculated as 0.7359 MJ/m^2^, 0.9276 MJ/m^2^, and 0.0739 MJ/m^2^, respectively. The *R*^2^ result of the algorithm is 0.8517. Given that all performance measurement benchmarks, it is noticed that the HHO algorithm produced the least accurate estimates for Ağrı province.

Figure 10c depicts the forecasted GSR data with the BMO algorithm for the Ağrı province. As shown in Table 4, the algorithm provided the *R*^*2*^ of 0.8681, MABE of 0.7164 MJ/m^2^, RMSE of 0.8845 MJ/m^2^, and MBE of 0.0371 MJ/m^2^ in the forecasting of GSR data for Ağrı province. Considering the *R*^2^ and RMSE metric results, it is seen that BMO is one of the two best algorithms that provided the most accurate prediction results together with GBO.

For Ağrı province, the predicted GSR data with the SCA algorithm is illustrated in Fig. 10d. As can be seen in Table 4, the *R*^2^, MABE, RMSE, and MBE results of the algorithm are 0.8652, 0.7113 MJ/m^2^, 0.8960 MJ/m^2^, and 0.0355 MJ/m^2^. The algorithm presented the lowest MBE value for the Ağrı province. The prediction accuracy of the SCA algorithm in terms of MABE metric results is better than BMO, HGSO, and HHO algorithms for Ağrı province.

The trend of GSR data forecasted by the HGSO algorithm for Ağrı province is displayed in Fig. 10e. As per the results in Table 4, HGSO yielded the second-best *R*^2^ result with a value of 0.8677 for Ağrı province. However, MABE metric results show that HGSO’s prediction accuracy is weaker than its competitors.

Overall, for the Ağrı province GBO algorithm achieved the best prediction results with an *R*^2^, MABE, and RMSE of 0.8810, 0.6703 MJ/m^2^, and 0.8432 MJ/m^2^, respectively. The SCA algorithm obtained the lowest MBE value of 0.0355 MJ/m^2^. HGSO is the only algorithm that calculates the MBE value negatively. This shows that the numerical value of GSR data estimated with HGSO for Ağrı province is larger than the measured data.

**Fig. 11:**
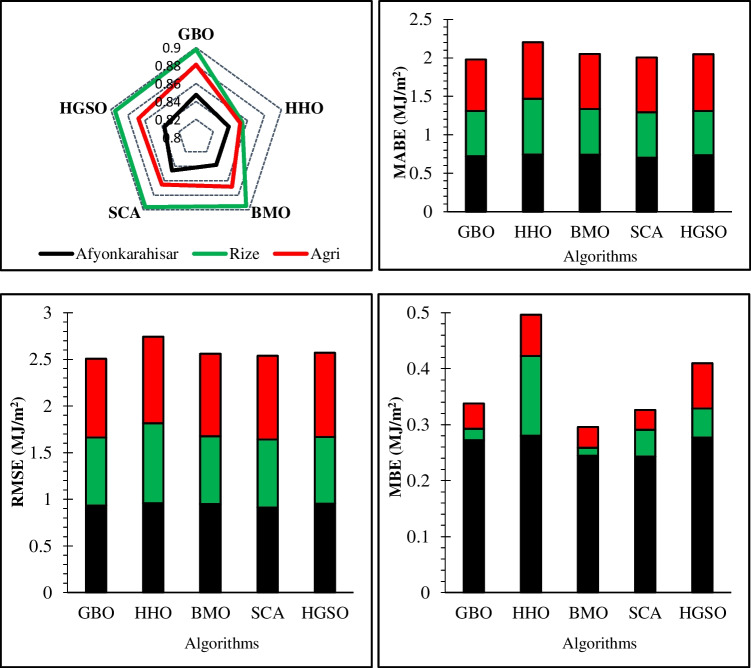
Visualization of statistical metric results

Figure 11 has been prepared to facilitate understanding of the data given in Table 4. As can be seen in the figure, the province –Rize- has the highest *R*^2^ value for each algorithm. It varies from 0.8543 to 0.8978 according to the algorithm. It is concluded that there is a strong correlation between input and solar radiation data for Rize province. In that province, algorithms exhibit better *R*^2^ results than in the Afyonkarahisar and Ağrı provinces. Nevertheless, the algorithms have very close and satisfying *R*^2^ values to that of Rize province in Afyonkarahisar and Ağrı provinces. Considering the *R*^2^ values in all algorithms cumulatively, bigger values are obtained for the GBO algorithm in each province, and the worst value is generally obtained for the HHO algorithm in each province. Upon examination of MABE results, it is seen that the HHO algorithm cumulatively has the highest MABE value for the prediction of daily GSR data. That is, the biggest errors are obtained for the HHO algorithm considering all provinces together. MABE values for the HHO algorithm are 0.7424 MJ/m^2^, 0.7265 MJ/m^2^, and 0.7359 MJ/m^2^ for Afyonkarahisar, Rize, and Ağrı provinces, respectively. On the other hand, the lowest MABE errors cumulatively are calculated for GBO, and its value is 0.7188 MJ/m^2^ for Afyonkarahisar, 0.5895 MJ/m^2^ for Rize, and 0.6703 MJ/m^2^ for Ağrı. Accordingly, it is possible to conclude that the MABE values for each algorithm in all provinces are very close to zero, which means that the error in predicting the solar radiation data is very satisfactory in terms of the MABE metric for each algorithm. Considering that all provinces are together, cumulatively the worst RMSE value is found for the HHO algorithm, while the best RMSE result is found for the GBO algorithm. As per the MBE metric results, it is seen that the HHO algorithm again has the worst result, but the best results are obtained for the BMO algorithm. For this metric, there are some negative results, and these results pull back the cumulative results of this metric for that algorithm. That is, the error magnitudes may be bigger for this metric, but when it is inverted, it can mislead the graph by showing the error magnitude cumulatively small. It will be reasonable that the better results are separately decided with the magnitude of the MBE metric. Accordingly, the worst result in all algorithms is found for Afyonkarahisar, while the lowest MBE values are generally found for Ağrı province, and it is very close to zero.

## Conclusions

This paper has focused on the prediction of daily global solar radiation data of three provinces (Afyonkarahisar, Rize, and Ağrı) in Türkiye using the GBO, HHO, BMO, SCA, and HGSO algorithms. The dataset includes historical data on sunshine duration, actual pressure, moisture, wind speed, and ambient temperature between 2010 and 2018 years. The performance success of metaheuristic algorithms is assessed with *R*^2^, MABE, RMSE, and MBE statistical metrics. The findings of the present study can be summarized as follows:

- In this work, GBO, HHO, BMO, SCA, and HGSO algorithms were used for GSR data estimation of Türkiye and applied to provinces selected from different regions to reflect the entire climatic conditions of the country. The algorithms generally gave high prediction accuracy, and this study is important in proving that these metaheuristic algorithms can also be used for GSR prediction.
- It was observed that the high accuracy of the GSR estimation results obtained could be considered an alternative solution, especially for regions where there is no GSR measurement device. Considering the high purchasing costs and maintenance expenses of GSR devices, it was understood that these algorithms could be used especially for regions where GSR data is needed.
- In Afyonkarahisar province, the SCA algorithm achieved the best prediction accuracy with MABE of 0.7023 MJ/m^2^, RMSE of 0.9121 MJ/m^2^, and MBE of 0.2430 MJ/m^2^.
- It is observed that GBO is the most fitting algorithm in the forecasting of daily GSR data for Ağrı province. The algorithm achieved the best forecasting accuracy with 0.8432 MJ/m^2^ of RMSE, 0.6703 MJ/m^2^ of MABE, and 0.8810 of *R*^2^.
- All algorithms obtained the highest *R*^2^ result in Rize province. *R*^2^ value varies from 0.8543 to 0.8978. Accordingly, it can be said that there is a strong correlation between inputs and solar radiation data for Rize province.
- Given that the prediction results of all provinces are together, it is noticed that HHO generally performed the lowest prediction accuracy among considered methods.

To sum up, it is noticed that there is not only one algorithm that accurately predicts the GSR data for any region. That is one algorithm, which gives the best GSR prediction result for a region, may give the worst results for another region. The performance of the algorithms is highly dependent on many factors such as the correlation of the input and output data, algorithms’ capabilities, and tuning of the parameters. In this case, it will be beneficial to test more algorithms for a region to decide on the best algorithm. From this point of view, GBO, HHO, BMO, SCA, and HGSO algorithms in the present work have offered satisfying results for the prediction of daily global solar radiation data in Afyonkarahisar, Rize, and Ağrı provinces. The statistical metric results showed that GBO and SCA algorithms predict more accurate solar radiation data compared to other algorithms used in this work. The superior performance of these two algorithms can be attributed to their ability to establish a good balance of exploration–exploitation, effectively explore the search space, and successfully imitate the process in nature. In the final, the authors encourage future works to test the algorithms used in this paper under varying environmental conditions and climate scenarios.

## References

[CR1] Ağbulut Ü (2022). A novel stochastic model for very short-term wind speed forecasting in the determination of wind energy potential of a region: a case study from Turkey. Sustain Energy Technol Assess.

[CR2] Ağbulut Ü, Gürel AE, Biçen Y (2021). Prediction of daily global solar radiation using different machine learning algorithms: evaluation and comparison. Renew Sustain Energy Rev.

[CR3] Ağbulut Ü, Yıldız G, Bakır H, Polat F, Biçen Y, Ergün A, Gürel AE (2023). Current practices, potentials, challenges, future opportunities, environmental and economic assumptions for Türkiye’s clean and sustainable energy policy: a comprehensive assessment. Sustain Energy Technol Assess.

[CR4] Agwa AM, Elsayed SK, Elattar EE (2022). Extracting the parameters of three-diode model of photovoltaics using barnacles mating optimizer. Symmetry.

[CR5] Ahmadianfar I, Bozorg-Haddad O, Chu X (2020). Gradient-based optimizer: a new metaheuristic optimization algorithm. Inf Sci.

[CR6] Ahmed S, Ghosh KK, Bera SK, Schwenker F, Sarkar R (2020). Gray level image contrast enhancement using barnacles mating optimizer. IEEE Access.

[CR7] Alrashidi M, Alrashidi M, Rahman S (2021). Global solar radiation prediction: application of novel hybrid data-driven model. Appl Soft Comput.

[CR8] Awasthi A, Shukla AK, SR MM, Dondariya C, Shukla KN, Porwal D, Richhariya G (2020). Review on sun tracking technology in solar PV system. Energy Rep.

[CR9] Bamisile O, Oluwasanmi A, Ejiyi C, Yimen N, Obiora S, Huang Q (2022). Comparison of machine learning and deep learning algorithms for hourly global/diffuse solar radiation predictions. Int J Energy Res.

[CR10] Belmahdi B, Bouardi AE (2024). Short-term solar radiation forecasting using machine learning models under different sky conditions: evaluations and comparisons. Environ Sci Pollut Res.

[CR11] Bhookya J, Jatoth RK (2019). Optimal FOPID/PID controller parameters tuning for the AVR system based on sine–cosine-algorithm. Evol Intel.

[CR12] Bounoua Z, Chahidi LO, Mechaqrane A (2021). Estimation of daily global solar radiation using empirical and machine-learning methods: a case study of five Moroccan locations. Sustain Mater Technol.

[CR13] Cannizzaro D, Aliberti A, Bottaccioli L, Macii E, Acquaviva A, Patti E (2021). Solar radiation forecasting based on convolutional neural network and ensemble learning. Expert Syst Appl.

[CR14] Colgan JD, Gard-Murray AS, Hinthorn M (2023). Quantifying the value of energy security: how Russia’s invasion of Ukraine exploded Europe's fossil fuel costs. Energy Res Soc Sci.

[CR15] Das S, Bhattacharya A, Chakraborty AK (2018). Solution of short-term hydrothermal scheduling using sine cosine algorithm. Soft Comput.

[CR16] Demir V, Citakoglu H (2023). Forecasting of solar radiation using different machine learning approaches. Neural Comput Appl.

[CR17] Duarte LCB, da Paixão MA, da Fé Bastos LF, Conterato FS (2022). Comparative between neural networks generate predictions for global solar radiation and air temperature. J Bioeng Technol Health.

[CR18] Fan J, Wang X, Wu L, Zhang F, Bai H, Lu X, Xiang Y (2018). New combined models for estimating daily global solar radiation based on sunshine duration in humid regions: a case study in South China. Energy Convers Manag.

[CR19] Fan J, Wu L, Zhang F, Cai H, Wang X, Lu X, Xiang Y (2018). Evaluating the effect of air pollution on global and diffuse solar radiation prediction using support vector machine modeling based on sunshine duration and air temperature. Renew Sustain Energy Rev.

[CR20] Fan J, Wu L, Zhang F, Cai H, Ma X, Bai H (2019). Evaluation and development of empirical models for estimating daily and monthly mean daily diffuse horizontal solar radiation for different climatic regions of China. Renew Sustain Energy Rev.

[CR21] Fan J, Wu L, Ma X, Zhou H, Zhang F (2020). Hybrid support vector machines with heuristic algorithms for prediction of daily diffuse solar radiation in air-polluted regions. Renew Energy.

[CR22] Feng Y, Hao W, Li H, Cui N, Gong D, Gao L (2020). Machine learning models to quantify and map daily global solar radiation and photovoltaic power. Renew Sustain Energy Rev.

[CR23] Geetha A, Santhakumar J, Sundaram KM, Usha S, Thentral TT, Boopathi CS, Ramya R, Sathyamurthy R (2022) Prediction of hourly solar radiation in Tamil Nadu using ANN model with different learning algorithms. Energy Rep 8:664–671

[CR24] Ghimire S, Nguyen-Huy T, Deo RC, Casillas-Perez D, Salcedo-Sanz S (2022). Efficient daily solar radiation prediction with deep learning 4-phase convolutional neural network, dual stage stacked regression and support vector machine CNN-REGST hybrid model. Sustain Mater Technol.

[CR25] Gianfreda A, Parisio L, Pelagatti M (2016) The impact of RES in the Italian day-ahead and balancing markets. Energy J 37(2_suppl):161–184

[CR26] Gouda SG, Hussein Z, Luo S, Yuan Q (2019). Model selection for accurate daily global solar radiation prediction in China. J Clean Prod.

[CR27] Guchua A, Jomidava M (2023) Energy security strategy of the European Union in the background of the Russia-Ukraine war. Futur Hum Image 20:46–54

[CR28] Guermoui M, Melgani F, Gairaa K, Mekhalfi ML (2020). A comprehensive review of hybrid models for solar radiation forecasting. J Clean Prod.

[CR29] Gürel AE, Ağbulut Ü, Biçen Y (2020). Assessment of machine learning, time series, response surface methodology and empirical models in prediction of global solar radiation. J Clean Prod.

[CR30] Hashim FA, Houssein EH, Mabrouk MS, Al-Atabany W, Mirjalili S (2019). Henry gas solubility optimization: a novel physics-based algorithm. Futur Gener Comput Syst.

[CR31] Hassan MH, Kamel S, El-Dabah MA, Rezk H (2021). A novel solution methodology based on a modified gradient-based optimizer for parameter estimation of photovoltaic models. Electronics.

[CR32] Heidari AA, Mirjalili S, Faris H, Aljarah I, Mafarja M, Chen H (2019). Harris hawks optimization: algorithm and applications. Futur Gener Comput Syst.

[CR33] Hoang AT, Nguyen XP (2021). Integrating renewable sources into energy system for smart city as a sagacious strategy towards clean and sustainable process. J Clean Prod.

[CR34] Huang L, Kang J, Wan M, Fang L, Zhang C, Zeng Z (2021). Solar radiation prediction using different machine learning algorithms and implications for extreme climate events. Front Earth Sci.

[CR35] IEA (2023) International energy agency [Online]. iea.blob.core.windows.net/assets/96d66a8b-d502-476b-ba94-54ffda84cf72/Renewables_2023.pdf

[CR36] Ismaeel AA, Houssein EH, Oliva D, Said M (2021). Gradient-based optimizer for parameter extraction in photovoltaic models. IEEE Access.

[CR37] Jamei M, Ahmadianfar I, Jamei M, Karbasi M, Heidari AA, Chen H (2022). Estimating daily global solar radiation in hot semi-arid climate using an efficient hybrid intelligent system. Eur Phys J Plus.

[CR38] Jathar LD, Nikam K, Awasarmol UV, Gurav R, Patil JD, Shahapurkar K, Soudagar MEM, Khan TMY, Kalam MA, Hnydiuk-Stefan A, Gürel AE, Hoang AT, Ağbulut Ü (2024) A comprehensive analysis of the emerging modern trends in research on photovoltaic systems and desalination in the era of artificial intelligence and machine learning. Heliyon 10:e25407 10.1016/j.heliyon.2024.e25407 PMC10873676 38371991

[CR39] Jiang H, Lu N, Qin J, Tang W, Yao L (2019). A deep learning algorithm to estimate hourly global solar radiation from geostationary satellite data. Renew Sustain Energy Rev.

[CR40] Jumin E, Basaruddin FB, Yusoff YB, Latif SD, Ahmed AN (2021). Solar radiation prediction using boosted decision tree regression model: a case study in Malaysia. Environ Sci Pollut Res.

[CR41] Kamboj VK, Nandi A, Bhadoria A, Sehgal S (2020). An intensify Harris Hawks optimizer for numerical and engineering optimization problems. Appl Soft Comput.

[CR42] Khosravi A, Koury RNN, Machado L, Pabon JJG (2018). Prediction of hourly solar radiation in Abu Musa Island using machine learning algorithms. J Clean Prod.

[CR43] Kıran MS, Özceylan E, Gündüz M, Paksoy T (2012). A novel hybrid approach based on particle swarm optimization and ant colony algorithm to forecast energy demand of Türkiye. Energy Convers Manag.

[CR44] Kouyakhi NR (2023). Exploring the interplay among energy dependence, CO2 emissions, and renewable resource utilization in developing nations: empirical insights from Africa and the middle east using a quantile-on-quantile approach and spatial analysis. Energy.

[CR45] Krane J, Idel R (2021). More transitions, less risk: how renewable energy reduces risks from mining, trade and political dependence. Energy Res Soc Sci.

[CR46] Liu JL, Fu J, Wong SS, Bashir S (2023) Energy security and sustainability for the European Union after/during the Ukraine Crisis: a perspective. Energy Fuels 37(5):3315–3327

[CR47] Marco-Lajara B, Martínez-Falcó J, Sánchez-García E, Millan-Tudela LA (2023). Analyzing the role of renewable energy in meeting the sustainable development goals: a bibliometric analysis. Energies.

[CR48] MENR (2022a) Republic of Türkiye ministry of energy and natural resources [Online]. https://enerji.gov.tr/eigm-yenilenebilir-enerji-kaynaklar-gunes#:~:text=%C3%9Clkemiz%2C%20co%C4%9Frafi%20konumu%20nedeniyle%20%C3%B6nemli,kWh%2Fm2%20olarak%20hesaplanm%C4%B1%C5%9Ft%C4%B1r. Accessed 12 Nov 2022

[CR49] MENR (2022b) Republic of Türkiye ministry of energy and natural resources January. 7:2020 [Online]. http://www.yegm.gov.tr/MyCalculator/. Accessed 23 Dec 2022

[CR50] Mirjalili S (2016). SCA: a sine cosine algorithm for solving optimization problems. Knowl-Based Syst.

[CR51] Mirza AF, Mansoor M, Ling Q (2020). A novel MPPT technique based on Henry gas solubility optimization. Energy Convers Manag.

[CR52] Mousavi SM, Mostafavi ES, Jiao P (2017). Next generation prediction model for daily solar radiation on horizontal surface using a hybrid neural network and simulated annealing method. Energy Convers Manag.

[CR53] Neggaz N, Houssein EH, Hussain K (2020). An efficient henry gas solubility optimization for feature selection. Expert Syst Appl.

[CR54] Nematchoua MK, Orosa JA, Afaifia M (2022). Prediction of daily global solar radiation and air temperature using six machine learning algorithms; a case of 27 European countries. Eco Inform.

[CR55] Nguyen XP, Le ND, Pham VV, Huynh TT, Dong VH, Hoang AT (2021) Mission, challenges, and prospects of renewable energy development in Vietnam. Energy sources, part a: recovery, utilization, and environmental effects 1–13. 10.1080/15567036.2021.1965264

[CR56] Nguyen VN, Tarełko W, Sharma P, El-Shafay AS, Chen WH, Nguyen PQP, Nguyen XP, Hoang AT (2024) Potential of explainable artificial intelligence in advancing renewable energy: challenges and prospects. Energy Fuels 38(3):1692–1712

[CR57] Pang Y, Zhang J, Ma R, Qu Z, Lee E, Luo T (2020). Solar–thermal water evaporation: a review. ACS Energy Lett.

[CR58] Patchali TE, Oyewola OM, Ajide OO, Matthew OJ, Salau TA, Adaramola MS (2022). Assessment of global solar radiation estimates across different regions of Togo, West Africa. Meteorol Atmos Phys.

[CR59] Patel D, Patel S, Patel P, Shah M (2022) Solar radiation and solar energy estimation using ANN and Fuzzy logic concept: a comprehensive and systematic study. Environ Sci Pollut Res 29(22):32428–32442 10.1007/s11356-022-19185-z 35178628

[CR60] Peñalva JJ, Lozano DA, Murillo JC, Ortega FM (2022) Global solar radiation time series forecasting using different architectures of the multilayer perceptron model. In: Journal of Physics: Conference Series. IOP Publishing 2180(1):012017

[CR61] Qiu R, Li L, Wu L, Agathokleous E, Liu C, Zhang B, Luo Y, Sun S (2022) Modeling daily global solar radiation using only temperature data: past, development, and future. Renew Sustain Energy Rev 163:112511

[CR62] Qu C, He W, Peng X, Peng X (2020). Harris hawks optimization with information exchange. Appl Math Model.

[CR63] Rizk-Allah RM, El-Fergany AA (2021). Effective coordination settings for directional overcurrent relay using hybrid Gradient-based optimizer. Appl Soft Comput.

[CR64] Rodríguez-Benítez FJ, Arbizu-Barrena C, Huertas-Tato J, Aler-Mur R, Galván-León I, Pozo-Vázquez D (2020). A short-term solar radiation forecasting system for the Iberian Peninsula. Part 1: models description and performance assessment. Sol Energy.

[CR65] Said Z, Sharma P, Tiwari AK, Huang Z, Bui VG, Hoang AT (2022). Application of novel framework based on ensemble boosted regression trees and Gaussian process regression in modelling thermal performance of small-scale Organic Rankine Cycle (ORC) using hybrid nanofluid. J Clean Prod.

[CR66] Sarwagya K, Nayak PK, Ranjan S (2020). Optimal coordination of directional overcurrent relays in complex distribution networks using sine cosine algorithm. Electric Power Syst Res.

[CR67] Shah D, Patel K, Shah M (2021). Prediction and estimation of solar radiation using artificial neural network (ANN) and fuzzy system: a comprehensive review. Int J Energy Water Resour.

[CR68] Sharma P, Said Z, Kumar A, Nizetic S, Pandey A, Hoang AT, Huang Z, Afzal A, Li C, Le AT, Nguyen XP, Tran VD (2022) Recent advances in machine learning research for nanofluid-based heat transfer in renewable energy system. Energy Fuels 36(13):6626–6658

[CR69] Soomar AM, Hakeem A, Messaoudi M, Musznicki P, Iqbal A, Czapp S (2022). Solar photovoltaic energy optimization and challenges. Front Energy Res.

[CR70] Srivastava R, Tiwari AN, Giri VK (2019). Solar radiation forecasting using MARS, CART, M5, and random forest model: a case study for India. Heliyon.

[CR71] Sulaiman MH, Mustaffa Z (2021). Solving optimal power flow problem with stochastic wind–solar–small hydro power using barnacles mating optimizer. Control Eng Pract.

[CR72] Sulaiman MH, Mustaffa Z, Saari MM, Daniyal H (2020). Barnacles mating optimizer: a new bio-inspired algorithm for solving engineering optimization problems. Eng Appl Artif Intell.

[CR73] Tao H, Ewees AA, Al-Sulttani AO, Beyaztas U, Hameed MM, Salih SQ, Armanuos AM, Al-Ansari N, Voyant C, Shadid S, Yaseen ZM (2021) Global solar radiation prediction over North Dakota using air temperature: development of novel hybrid intelligence model. Energy Rep 7:136–157

[CR74] Tefek MF, Uğuz H, Güçyetmez M (2019). A new hybrid gravitational search–teaching–learning-based optimization method for energy demand estimation of Türkiye. Neural Comput Appl.

[CR75] TSMS (2022) Turkish State Meteorological Service. (January 7, 2020). https://mgm.gov.tr/eng/

[CR76] Woldegiyorgis TA, Admasu A, Benti NE, Asfaw AA (2022). A comparative evaluation of artificial neural network and sunshine based models in prediction of daily global solar radiation of Lalibela, Ethiopia. Cogent Eng.

[CR77] Xin B, Zhang M (2023). Evolutionary game on international energy trade under the Russia-Ukraine conflict. Energy Econ.

[CR78] Yang L, Cao Q, Yu Y, Liu Y (2020). Comparison of daily diffuse radiation models in regions of China without solar radiation measurement. Energy.

[CR79] Zang H, Cheng L, Ding T, Cheung KW, Wang M, Wei Z, Sun G (2020). Application of functional deep belief network for estimating daily global solar radiation: a case study in China. Energy.

[CR80] Zhang Y, Cui N, Feng Y, Gong D, Hu X (2019). Comparison of BP, PSO-BP and statistical models for predicting daily global solar radiation in arid Northwest China. Comput Electron Agric.

[CR81] Zhang Y, Zhou X, Shih PC (2020). Modified Harris Hawks optimization algorithm for global optimization problems. Arab J Sci Eng.

[CR82] Zhou Y, Liu Y, Wang D, Liu X, Wang Y (2021). A review on global solar radiation prediction with machine learning models in a comprehensive perspective. Energy Convers Manag.

